# High-Resolution CSRR-Based Microwave Sensor for Soil Moisture Content Monitoring

**DOI:** 10.3390/s26175665

**Published:** 2026-09-06

**Authors:** Salman Alduwish, Yongxiang Li, James Scott, Akram Hourani, Nasir Mahmood

**Affiliations:** 1 School of Engineering, RMIT University, Melbourne, VIC 3000, Australia; yongxiang.li@rmit.edu.au (Y.L.); james.scott@rmit.edu.au (J.S.); akram.hourani@unimelb.edu.au (A.H.); 2 School of Science, RMIT University, Melbourne, VIC 3000, Australia; nasir.mahmood@rmit.edu.au

**Keywords:** CSRR, microwave sensor, soil moisture, machine learning, precision agriculture

## Abstract

This work presents a compact square complementary split-ring resonator (CSRR) microwave sensor, combined with a machine-learning-based calibration strategy, to achieve superior texture-aware soil moisture quantification. Implemented on a Rogers RO3010 substrate with a 20 × 30 mm^2^ footprint and operating near 1.3 GHz, the sensor exploits shifts in resonance/notch frequency and insertion loss (S21) to probe both the real and imaginary components of the soil’s complex permittivity. Full-wave 3D electromagnetic simulations guided optimisation of the CSRR topology and T-shaped microstrip feedline, yielding strong field confinement, high quality factor, and high Frequency Detection Resolution (FDR). Experiments on sand and loam across 0–30% and 0–40% moisture content ranges, respectively, demonstrate FDR values of 6.09 MHz (sand) and 6.86 MHz (loam), enabling discrimination of subtle permittivity changes. Several calibration strategies are developed and compared for complex permittivity extraction from measured S-parameters: linear and polynomial regression, a multivariable least-squares sensitivity-matrix model, and a delta-referenced multilayer perceptron (MLP) with z-score standardization. While polynomial and least-squares models significantly outperform linear regression (R^2^ > 0.997), the MLP combined with the optimized CSRR architecture delivers the best performance, achieving near-ideal accuracy (R^2^ ≈ 1, MAE < 0.001, RMSE < 0.001) for both soil types. These results demonstrate that the synergy between the novel CSRR sensor design and data-driven MLP calibration enables high-resolution, robust, and field-deployable soil moisture sensing, offering a compelling solution for next-generation agricultural and geotechnical monitoring systems.

## 1. Introduction

Soil moisture is a key state variable in the Earth system, governing water and energy exchanges between the land surface and atmosphere, and directly influencing crop yield, irrigation efficiency, and slope stability in geotechnical structures. Reliable, spatially resolved soil moisture information underpins precision agriculture, climate-resilient farming, and infrastructure safety by enabling data-driven decisions on irrigation scheduling, fertilizer application, and soil reinforcement strategies.

Because water has a much higher permittivity than dry soil minerals and air, the complex dielectric permittivity of soil is a robust proxy for its moisture content. This has motivated the development of dielectric-based sensing techniques, where soil moisture is inferred from the interaction between an electromagnetic field and the soil matrix [1,2,3,4]. Among these, microwave sensors have attracted particular interest due to their ability to operate non-invasively, provide rapid measurements, and achieve high sensitivity to permittivity variations. By tracking shifts in resonance frequency and scattering parameters, microwave resonant structures transduce changes in soil water content into measurable electromagnetic signatures.

Within this class, complementary split-ring resonator (CSRR)-based planar sensors offer several advantages: a compact footprint, high quality factor, and strong electromagnetic field confinement, which together enhance sensitivity to small permittivity changes in the near-field region [5,6,7,8,9,10,11,12]. These properties make CSRR sensors promising candidates for embedded and field-deployable soil moisture monitoring. However, accurate moisture quantification from CSRR responses remains challenging. The relationship between soil water content and complex permittivity is highly nonlinear and soil-specific, influenced by texture, bulk density, temperature, and salinity. Moreover, most existing CSRR-based soil sensors primarily exploit resonance frequency shifts linked to the real part of permittivity, giving limited consideration to the imaginary part associated with dielectric losses and attenuation. As a result, many empirical calibration approaches—typically linear or low-order polynomial fits—struggle to generalize across soil types and moisture ranges, and often neglect the full information content available in the sensor’s S-parameters [13,14,15,16,17,18].

Conventional dielectric soil moisture sensors such as time-domain reflectometry (TDR), time-domain transmissometry (TDT), and frequency-domain reflectometry (FDR) probes are widely used because they provide relatively robust volumetric moisture estimates over sensing lengths of several centimeters to decimeters and have well-established calibration procedures [1,2,3,4,13,14,15]. However, their physical size and extended measurement volume can make them less suitable for highly localized, near-surface, or layered investigations, and their installation may disturb the soil structure and root environment. Recently reported planar microwave and metamaterial-inspired sensors (including DGS, CSRR, and fractal resonators) offer more compact form factors and high sensitivity to permittivity changes in the near field [5,6,7,8,9,10,11,12,19,20,21,22], but their smaller electromagnetic penetration depth and limited lateral extent imply a substantially smaller “volume of influence” compared with conventional TDR/TDT/FDR probes.

In this context, the proposed square CSRR-based sensor is positioned as complementary rather than as a direct replacement for established volumetric probes. Its 20 × 30 mm^2^ footprint and strong field confinement enable high-resolution, texture-aware characterization of soil permittivity in a well-controlled, localized sensing zone, which is advantageous for applications such as small soil columns, lysimeters, root-zone studies, or calibration of remote-sensing models. At the same time, the relatively small sensing volume means that, in structurally heterogeneous field soils, a single sensor may not be fully representative of the bulk moisture state. This limitation is now explicitly acknowledged, and quantitative characterization of the sensor’s three-dimensional volume of influence, as well as strategies to improve field-scale representativeness (e.g., sensor arrays or hybrid deployment with larger-volume TDR/TDT/FDR probes), are identified as important directions for future work rather than claims of the present study.

Recent advances have begun to address these limitations by incorporating multivariable analytical models and machine learning. Sensitivity-matrix approaches leverage both frequency and magnitude changes to recover complex permittivity, while multilayer perceptron (MLP) models and other data-driven techniques learn nonlinear mappings between sensor outputs and soil dielectric properties from experimental data [16,17,18]. Nevertheless, there is still a need for compact CSRR-based sensors that simultaneously (i) achieve high frequency detection resolution and sensitivity over practical moisture ranges for different soil textures, and (ii) integrate advanced calibration frameworks capable of accurately extracting both real and imaginary permittivity components from measured S-parameters.

This study addresses these gaps by designing, modelling, and experimentally validating a compact square CSRR-based microwave sensor implemented on a Rogers RO3010 substrate, operating near 1.3 GHz within a 20 × 30 mm^2^ footprint. The sensor is configured to detect moisture-induced variations in sand and loam soils through coupled shifts in resonance/notch frequency and insertion-loss magnitude (S21), thereby probing both the real and imaginary parts of the complex permittivity. Building on this hardware platform, we develop and compare multiple calibration strategies—linear and polynomial regression, a multivariable least-squares sensitivity-matrix method, and a machine learning-based MLP—to extract complex permittivity from measured sensor responses. The objectives are to (i) quantify the sensor’s frequency detection resolution and sensitivity for different soils, (ii) evaluate the accuracy of each calibration model in recovering real and imaginary permittivity over practical moisture ranges, and (iii) demonstrate that combining a high-resolution CSRR sensor with data-driven calibration enables robust, field-deployable soil moisture quantification for precision agriculture and geotechnical monitoring.

The organization of this paper is as follows: Section 2 describes the design and electromagnetic modelling of the compact square CSRR-based microwave sensor, including its geometrical configuration, operating principle, and simulated S-parameter response. Section 3 details the soil moisture estimation methodology, outlining the experimental setup, data acquisition procedure for sand and loam samples, and the empirical and data-driven calibration techniques (linear and polynomial fitting, least-squares sensitivity matrix, and MLP-based machine learning) used to extract complex permittivity. Section 4 presents the performance evaluation, including frequency detection resolution, sensitivity analysis, and a comparative assessment of the different calibration models, as well as a benchmark against existing microwave soil moisture sensors. Finally, Section 5 summarizes the main findings, discusses practical implications for precision agriculture and geotechnical monitoring, and highlights directions for future research.

## 2. Sensor Design and Modeling

**Figure 1:**
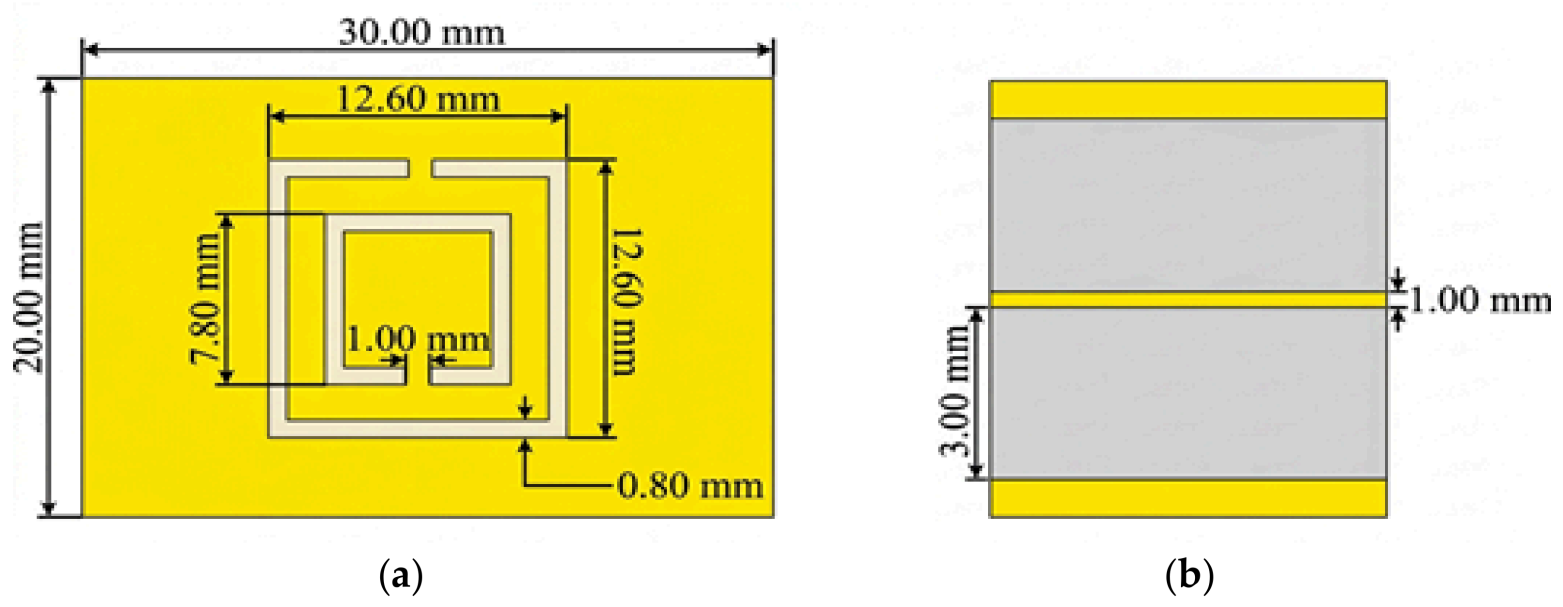
The proposed square CSRR sensor: (**a**) bottom view, and (**b**) top view.

**Figure 2:**
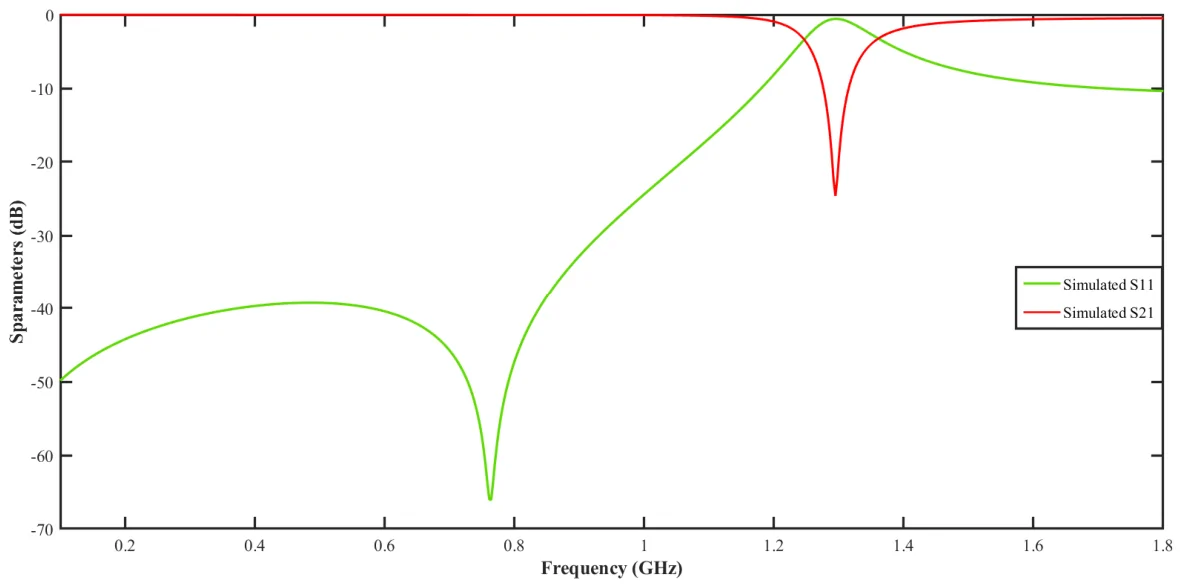
Simulated S_11_ and S_21_ response of the proposed CSRR sensor.

The proposed sensor is a compact square Complementary Split-Ring Resonator (CSRR)-based microwave sensor designed to characterize the dielectric properties of soil, which are directly influenced by soil moisture content. Fabricated on a Rogers RO3010 substrate, the sensor operates around 1.3 GHz within a 20 × 30 mm^2^ footprint, making it suitable for field deployment. Its resonance frequency and insertion loss (S21) responses are highly sensitive to changes in the soil’s relative permittivity, enabling precise detection of moisture variations. Figure 1 and Figure 2 illustrate key aspects of the proposed square CSRR sensor design and its simulated response.

The sensor response shown in Figure 2 was obtained using full-wave three-dimensional electromagnetic (EM) simulation of the complete physical layout. The square CSRR pattern, the microstrip feedline, and the Rogers RO3010 substrate (with manufacturer-specified relative permittivity and loss tangent) were modeled in CST Microwave Software. Wave ports were assigned at the microstrip input and output, and open (radiation) boundary conditions were applied to emulate free-space operation.

In this framework, the simulated S-parameters (S11, S21) directly capture the electromagnetic behavior of the realized geometry, including field confinement within and around the CSRR slots, coupling to the feedline, and substrate losses. These simulations were used to optimize the CSRR dimensions and feedline configuration to achieve a well-defined notch around 1.3 GHz with high quality factor and strong sensitivity to dielectric perturbations above the resonator.

The square CSRR topology was selected because its compact planar geometry facilitates easy integration in space-constrained field environments while providing strong electromagnetic field confinement. This confinement results in high sensitivity of both resonance frequency and insertion loss to subtle changes in the soil’s dielectric properties. Operating near 1.3 GHz provides a suitable trade-off between penetration depth in soil and sensitivity to permittivity variations: higher frequencies would experience stronger attenuation in moist soils, whereas significantly lower frequencies would reduce spatial resolution and increase sensor size. The use of Rogers RO3010 substrate further enhances performance by offering stable, well-characterized dielectric properties, leading to reproducible resonance behavior and reliable calibration.

The EM-simulated S-parameters confirm that the proposed square CSRR design achieves high Frequency Detection Resolution (FDR) and sensitivity, which are essential for resolving small changes in soil moisture over practical ranges for different soil types such as sand and loam. These simulated responses guided the final geometry optimization prior to fabrication, ensuring that the measured resonance frequency shifts and insertion-loss changes can be robustly exploited as primary observables in the empirical, least-squares, and machine learning-based calibration models developed in this work.

## 3. Soil Moisture Monitoring

### 3.1. Empirical Modeling Techniques for Extracting Sand Soil Complex Permittivity

Dielectric characterization of soil is crucial in precision agriculture and geotechnical engineering. The resonance frequency and insertion loss of a CSRR-based microwave sensor are strongly dependent on the relative permittivity of the tested soil samples.

To assess the sensor’s suitability before developing calibration models, two key performance metrics are evaluated: Frequency Detection Resolution (FDR) and sensitivity.

In this work, we define the Frequency Detection Resolution (FDR) as the average frequency–permittivity sensitivity over the investigated permittivity range. It is calculated as:

(1) $$FDR=\frac{f_{n_{u}}-f_{n_{l}}}{{\varepsilon_{r}}_{2}-{\varepsilon_{r}}_{1}}(GHz)$$

where $f_{n\_u}$ represents the highest resonant frequency, $f_{n\_l}$ represents the lowest resonant frequency, ${\varepsilon_{r}}_{2}$ represents the highest permittivity value of the soil sample, and ${\varepsilon_{r}}_{1}$ represents the lowest permittivity value of the soil sample.

The proposed sensor demonstrates an FDR of 6.09 MHz. This indicates a strong resolution capability, allowing the sensor to accurately isolate subtle variations in soil moisture.

**Figure 3:**
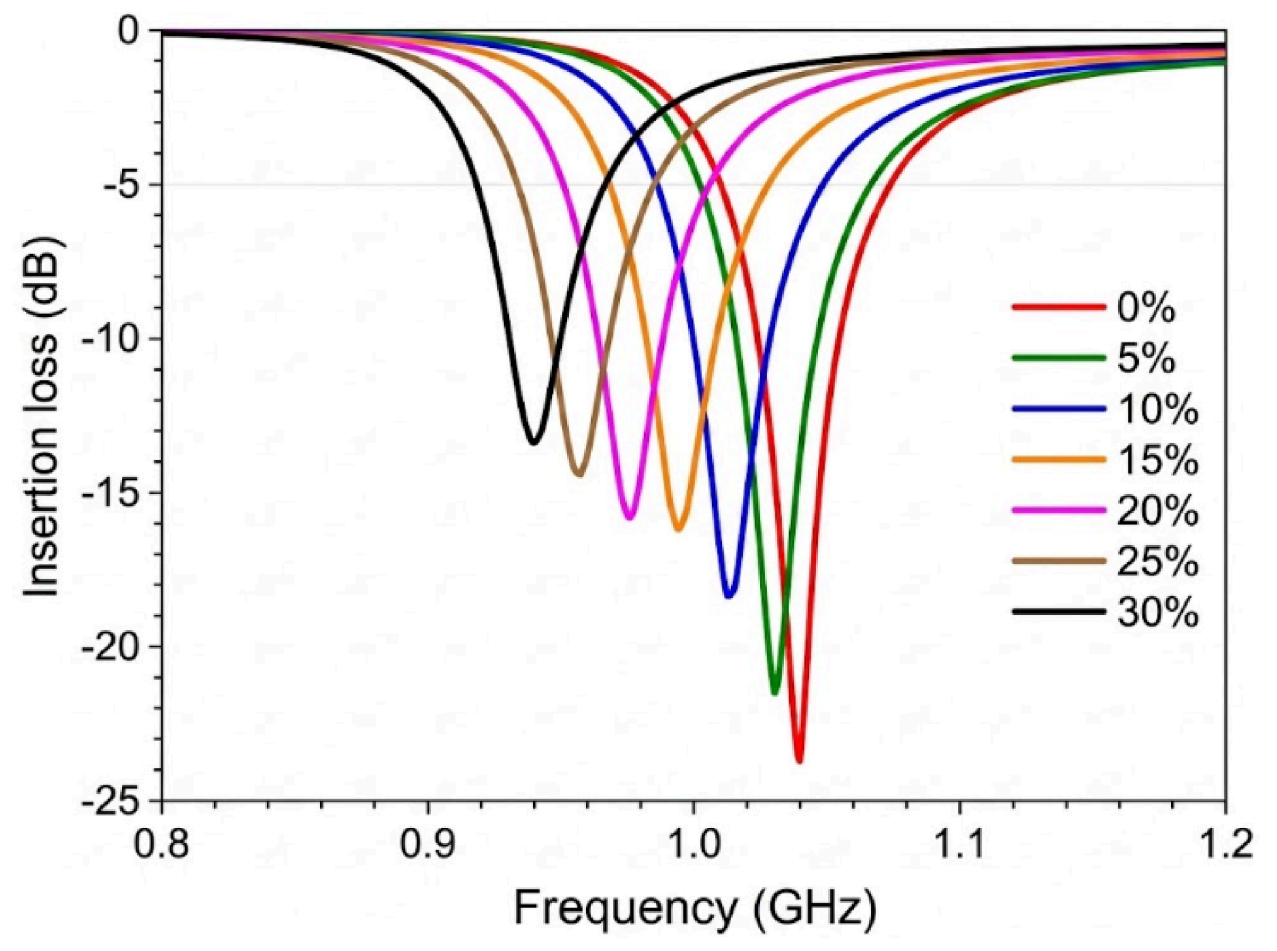
S_21_ frequency shifts with various moisture levels of sand soil samples.

To evaluate the performance of the designed square CSRR-based sensor (Figure 3), it is essential to analyze its sensitivity. This involves obtaining the variation in resonance frequency, which allows us to determine the sensor’s sensitivity.

(2) $$S_{n}=\frac{{∆f}_{notch}}{f_{notch\_empty}\times \left(\varepsilon^{\prime }-1\right)}\times 100\%$$

where ${∆f}_{notch}$ represents the notch frequency of the proposed MW sensor under loaded conditions (with the soil box), and $f_{notch\_empty}=1.3\;GHz$. The detailed simulated resonance frequencies, insertion losses, and corresponding sensitivities for different sand moisture levels are summarized in Table 1.

A numerical model is required to characterize the permittivity of the tested materials from measured physical parameters. Using curve-fitting techniques, the equation for the material permittivity is extracted directly from the measured scattering data.

For any given moisture content (MC) level, the absolute prediction error is calculated using the following equation:

(3) $$Error\;\left(\%\right)=\left|\frac{{\varepsilon_{simulated}}^{\prime }-{\varepsilon_{Dataset}}^{\prime }}{{\varepsilon_{Dataset}}^{\prime }}\right|\times 100$$

#### 3.1.1. Linear and Polynomial Fitting Model of Sand Soil 

**Figure 4:**
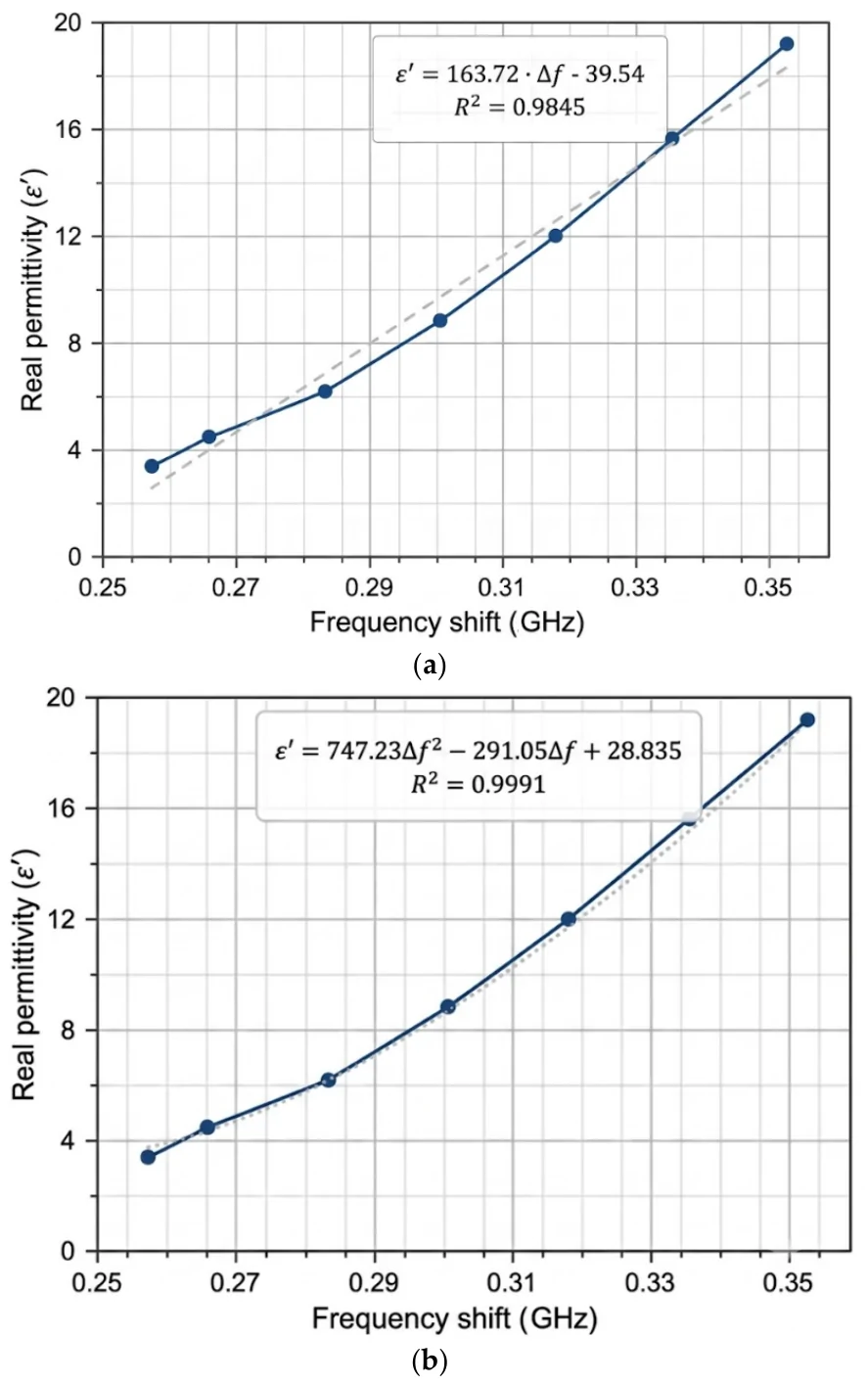
Curve fitting for real permittivity vs. frequency shift: (**a**) linear model, and (**b**) polynomial model.

Figure 4 shows the curve fitting of the relationship between the real permittivity and the resonance frequency shift for different soil moisture levels. In Figure 4a, a linear regression model is applied, assuming a first-order dependence of permittivity on the frequency shift. In contrast, Figure 4b presents a second-order polynomial fit, which allows for a more flexible, nonlinear relationship between these variables. The polynomial order was selected using a leave-one-out cross-validation strategy on the seven available sand calibration points: while moving from the linear model (first order) to a second-order polynomial markedly reduced the validation error, higher-order polynomials (order ≥ 3) increased the cross-validated error and produced oscillatory behavior between calibration points, indicating overfitting. Accordingly, a second-order polynomial was adopted as the final calibration model for sand.

Table 2 summarizes the fitting results for both models across the considered moisture contents (0–30% MC). For each moisture level, the table lists the corresponding resonance frequency (in GHz), the simulated real permittivity, and the values predicted by the linear and polynomial models. As can be observed, both models follow the general trend of increasing permittivity with increasing moisture content. However, the polynomial model provides estimates that are closer to the simulated values over the entire range, particularly at higher moisture contents, indicating an improved fitting accuracy compared with the linear approximation. This justifies the use of the polynomial model for calibration of the sensor response to soil moisture.

#### 3.1.2. Least-Squares Model for Sand Soil

To extract the complete dielectric profile—both real (ε′) and imaginary (ε″) permittivity—a multi-variable analytical model is required. This utilizes both the resonant frequency shift (Δf) and the change in insertion loss ($\Delta S_{21}$) [24,25,26].

This relationship is mathematically modeled using a sensitivity matrix (M):

(4) $$\left(\begin{array}{c} ∆f\\ \Delta S_{21} \end{array}\right)=\left(\begin{array}{cc} m_{11} & m_{12}\\ m_{21} & m_{22} \end{array}\right)\left(\begin{array}{c} ∆\varepsilon \prime \\ ∆\varepsilon \prime \prime \end{array}\right)$$

where coefficients $m_{ij}$ are sensor-dependent parameters determined by initial calibration using reference samples with known complex permittivity values. This methodology allows for the comprehensive extraction of the complex relative permittivity.

The relationships are defined as:

(5) $$\;\left\{\begin{array}{c} ∆\varepsilon \prime =\varepsilon \prime -\varepsilon_{ref}\prime \;;\;∆\varepsilon \prime \prime =\varepsilon \prime \prime -\varepsilon_{ref}\prime \prime \\ ∆f=f_{notch}-f_{notch\_ref}\;;\;\Delta S_{21}=S_{21}-S_{21\_ref}\; \end{array}\right.$$

The least-squares method can be used to approximate the unknown coefficients. Three matrices are required to approximate the unknown coefficients:

(6) $$X=\left(\begin{array}{cc} {∆\varepsilon^{\prime }}_{1} & {∆\varepsilon^{\prime \prime }}_{1}\\ {∆\varepsilon^{\prime }}_{2} & {∆\varepsilon^{\prime \prime }}_{2}\\ {∆\varepsilon^{\prime }}_{3} & {∆\varepsilon^{\prime \prime }}_{3}\\ {∆\varepsilon^{\prime }}_{4} & {∆\varepsilon^{\prime \prime }}_{4}\\ {∆\varepsilon^{\prime }}_{5} & {∆\varepsilon^{\prime \prime }}_{5}\\ {∆\varepsilon^{\prime }}_{6} & {∆\varepsilon^{\prime \prime }}_{6}\\ {∆\varepsilon^{\prime }}_{7} & {∆\varepsilon^{\prime \prime }}_{7} \end{array}\right);Y1=\left(\begin{array}{c} {∆f}_{1}\\ {∆f}_{2}\\ {∆f}_{3}\\ {∆f}_{4}\\ {∆f}_{5}\\ {∆f}_{6}\\ {∆f}_{7} \end{array}\right);Y2=\left(\begin{array}{c} {\Delta S_{21}}_{1}\\ {\Delta S_{21}}_{2}\\ {\Delta S_{21}}_{3}\\ {\Delta S_{21}}_{4}\\ {\Delta S_{21}}_{5}\\ {\Delta S_{21}}_{6}\\ {\Delta S_{21}}_{7} \end{array}\right)$$

The unknown coefficients can be calculated from:

(7) $$\left\{\begin{array}{c} {\left[\begin{array}{cc} m_{11} & m_{12} \end{array}\right]}^{T}={\left(X^{T}\;X\right)}^{-1}\;X^{T}\;Y1\\ {\left[\begin{array}{cc} m_{21} & m_{22} \end{array}\right]}^{T}={\left(X^{T}\;X\right)}^{-1}\;X^{T}\;Y2 \end{array}\right.$$

##### Data and Matrix Formulation

The primary objective is to establish a robust numerical model that correlates physical sensor responses—specifically resonance frequency shift ($∆f_{soil}$) and peak attenuation shift ($\Delta S_{21}$)—with the complex permittivity ($∆\varepsilon^{\prime }$, $∆\varepsilon^{\prime \prime }$) of the soil samples.

The calibration data used to construct the sandy soil sensor output model, including resonance frequency shifts and insertion-loss variations across moisture levels, are summarized in Table 3.

The corresponding input calibration data for the sandy soil sensor, including absolute resonance frequencies, insertion losses, and their shifts relative to the reference condition, are summarized in Table 4.

The model is built using seven data points corresponding to moisture levels from 0% to 30%. The 15% moisture level is designated as the reference point. All values in the matrices represent the change (∆) relative to this baseline.

The $\left[X\right]$ matrix (7 × 2) contains the independent variables: the change in the real part (∆ε′) and imaginary part (∆ε″) of permittivity.

$$X=\left(\begin{array}{cc} -5.4 & -0.6\\ -4.3 & -0.5\\ -2.6 & -0.3\\ 0 & 0\\ 3.2 & 0.1\\ 6.8 & 0.4\\ 10.3 & 0.7 \end{array}\right)$$

$\;\left[Y1\right]$ represents the measured frequency shifts (GHz), and $\left[Y2\right]$ represents the peak attenuation shifts (dB).

$$Y1=\left[\begin{array}{c} 0.0435\\ 0.0348\\ 0.0174\\ 0\\ -0.0174\\ -0.0348\\ \begin{array}{c} -0.0522 \end{array} \end{array}\right];\;Y2=\left[\begin{array}{c} 7.6\\ 5.388\\ 2.177\\ 0\\ -0.351\\ -1.76\\ \begin{array}{c} -2.784 \end{array} \end{array}\right]$$

##### Mathematical Derivation

Step 3.1: The sensitivity matrix $\left[M\right]$ is defined by the relationship: Y = X M. To solve for $\left[M\right]$, we use the normal equation: $M\;=\;{\left(X^{T}\;X\right)}^{-1}\;X^{T}\;Y$.

This step aggregates the variance and covariance of the dielectric properties across all test samples.

$$X^{T}X=\left[\begin{array}{cc} \sum {\left(∆\varepsilon \prime \right)}^{2} & \sum \left(∆\varepsilon \prime ∆\varepsilon \prime \prime \right)\\ \sum \left(∆\varepsilon \prime ∆\varepsilon \prime \prime \right) & \sum {\left(∆\varepsilon \prime \prime \right)}^{2} \end{array}\right]=\left[\begin{array}{cc} 216.98 & 16.42\\ 16.42 & 1.36 \end{array}\right]$$

Step 3.2: Inversion of the Covariance Matrix ${\left(X^{T}\;X\right)}^{-1}$.

To isolate the sensitivity coefficients, we must invert $X^{T}\;X$.

$${{(X}^{T}X)}^{-1}=\left[\begin{array}{cc} 0.05338 & -0.64452\\ -0.64452 & 8.51689 \end{array}\right]$$

Step 3.3: Correlation of Sensor Response $X^{T}\;Y$.

We calculate the cross-product of the dielectric changes and the sensor’s physical shifts:

For frequency:

$$X^{T}Y1=\left[\begin{array}{c} -1.25976\\ -0.10092 \end{array}\right]$$

For attenuation:

$$X^{T}Y2=\left[\begin{array}{c} -111.635\\ -10.595 \end{array}\right]$$

##### The Sensitivity Matrix (*M*)

Multiplying the inverse matrix by the correlation vectors yields the final sensitivity coefficients:

$$M=\left[\begin{array}{cc} m_{11} & m_{12}\\ m_{21} & m_{22} \end{array}\right]=\left[\begin{array}{cc} -0.0022 & 0.8694\\ -0.04763 & -18.28308 \end{array}\right]$$

##### Prediction Equations

To enable the sensor to “predict” the material properties of an unknown sample, we invert the sensitivity matrix M. This allows the user to input measured shifts of frequency and output dielectric changes.

The inversion of $\left[M\right]$ results in the following operational equations:

- Predicting Real Permittivity Change (∆ε′)

$$∆\varepsilon^{\prime }=-223.8669∆f+0.5826{∆S}_{21}$$

$$∆\varepsilon^{\prime }=\varepsilon^{\prime }-{\varepsilon^{\prime }}_{ref}$$

- Predicting Real Permittivity (ε′)

$$\varepsilon^{\prime }=-223.8669∆f+{0.5826∆S}_{21}+8.8$$

- Predicting Imaginary Permittivity Change (∆ε″)

$$∆\varepsilon^{\prime \prime }=-10.64∆f-0.027{∆S}_{21}$$

$$∆\varepsilon^{\prime \prime }=\varepsilon^{\prime \prime }-{\varepsilon^{\prime \prime }}_{ref}$$

- Predicting Imaginary Permittivity ($\varepsilon^{\prime \prime }$)

$$\varepsilon^{\prime \prime }=-10.64∆f-0.027{∆S}_{21}+0.6$$

The performance of the least-squares calibration model for sandy soil, including predicted real and imaginary permittivity components across moisture levels, is summarized in Table 5.

#### 3.1.3. Machine Learning Model for Sand Soil

The MLP model was implemented in Matlab 2024a using a multilayer perceptron with two inputs, a five-neuron hidden layer using a hyperbolic tangent activation function, and a two-neuron linear output layer. For data splitting, the model allocates 100% of the nine-sample dataset entirely to training, holding nothing back for validation or testing. Since no optimizer is explicitly declared, the network defaults to MATLAB’s Levenberg–Marquardt backpropagation algorithm, which relies on a dynamically adjusting damping factor rather than a fixed learning rate. The model is set to train for a maximum of 2000 epochs using the default Mean Squared Error loss function, configured to stop early if it reaches a strict performance goal of 1 × 10^−5^. Because the script evaluates the network on the exact same normalized dataset it was trained on, the final outputs represent only training errors; the code does not provide any independent test or cross-validation results to demonstrate how well the model generalizes to unseen data.

##### Data Preparation and Baseline Reference

We adopt a “delta” representation for the input features. By defining the 15% MC point as the reference operating condition and mapping it to (0, 0), the network is trained on relative variations of the material properties around this baseline. This centering of the data improves the numerical conditioning of the learning problem and typically facilitates faster and more stable convergence during training [27,28].

##### Input Normalization (Standardization)

Raw data from microwave sensors often has disparate scales—frequency shifts are small decimals, while magnitude shifts can be large integers.

We apply Z-score standardization:

(8) xnorm=(x−μ)σ

where

(9) $$\mu =\frac{\sum_{i=1}^{N}x_{i}}{N},\sigma =\sqrt{\frac{\sum {\left(x_{i}-\mu \right)}^{2}}{N}}$$

Values for ${∆f}_{soil}$: Mean μ=−0.001243  and scale σ=0.033024, and values for $\;∆S_{21}$: Mean μ=1.467143 and scale σ=3.534196.

$$\left\{\begin{array}{c} {x1}_{norm}=\frac{∆f+0.001243}{0.033024}\\ {x2}_{norm}=\frac{∆S_{21}-1.467143\;}{3.534196} \end{array}\right.$$

##### Neural Network Architecture

The model is a multilayer perceptron (MLP) [29,30] consisting of: Input Layer: two neurons (∆f, $∆S_{21}$), Hidden Layer: five neurons using the Hyperbolic Tangent (tanh) activation function. This handles the non-linear relationship between moisture and permittivity, and Output Layer: two neurons (ε′, ε″) using a linear transfer function.

The expressions for the hidden neurons $h_{k}$ (k=(1,…, 5) follow directly from the standard MLP formulation: each hidden neuron computes an affine combination of the two normalised inputs, followed by the chosen nonlinearity ($\tanh\left(\cdot \right)$). Mathematically:

(10) $$\;\left\{\begin{array}{c} z_{k}={\omega_{k1}}^{\left(1\right)}∆f+{\omega_{k2}}^{\left(1\right)}∆\left|S_{21}\right|+{b_{k}}^{\left(1\right)}\\ h_{k}=\tanh\left(z_{k}\right),\;k=1,\ldots ,\;5 \end{array}\right.$$

where ${\omega_{k1}}^{\left(1\right)}$ and ${\omega_{k2}}^{\left(1\right)}$ are the weights connecting the two input neurons to hidden neuron, ${b_{k}}^{\left(1\right)}$ is the bias term of hidden neuron k, $z_{k}$ is the pre-activation (linear) combination for neuron k, and $\tanh\left(.\right)$ is the activation function introducing nonlinearity.

These equations are the standard forward-propagation equations of a one-hidden-layer neural network with two inputs and five hidden units.

The two output neurons then form linear combinations of the hidden activations:

(11) $$\left\{\begin{array}{c} ∆\varepsilon \prime =\sum_{K=1}^{5}{\omega_{1k}}^{\left(2\right)}h_{k}+{b_{1}}^{\left(2\right)}\\ ∆\varepsilon \prime \prime =\sum_{K=1}^{5}{\omega_{2k}}^{\left(2\right)}h_{k}+{b_{2}}^{\left(2\right)} \end{array}\right.$$

where ${\omega_{mk}}^{\left(2\right)}$ are the weights from hidden neuron “k” to output neuron “m” (m = 1 for ∆ε′, m = 2 for ∆ε″), and ${b_{m}}^{\left(2\right)}$ are the corresponding output biases.

Finally, the predicted absolute permittivities are recovered by adding back the reference values:

(12) $$\;\left\{\begin{array}{c} \varepsilon^{\prime }={\varepsilon^{\prime }}_{ref}+∆\varepsilon^{\prime }\\ \varepsilon^{\prime \prime }={\varepsilon^{\prime \prime }}_{ref}+∆\varepsilon^{\prime \prime } \end{array}\right.$$

##### Prediction Equations

After convergence, the resulting optimized weights and biases constitute the numerical coefficients appearing in the prediction equations. In other words, the expressions for the hidden neurons $h_{1}\;$ to $h_{5\;}\;$ are fixed by the chosen network structure (affine transformation plus tanh).

$$\left\{\begin{array}{c} h1=tanh(\;(\;0.3178\;*\;x1\_norm\;)-(0.1476\;*\;x2\_norm)-0.2538\;)\\ h2=tanh(\;(\;5.6653\;*\;x1\_norm)+(\;1.1276\;*\;x2\_norm)-3.9336\;)\\ h3=tanh(\;(\;2.7366\;*\;x1\_norm\;)-(2.0939\;*\;x2\_norm)+0.6576\;)\\ h4=tanh(\;(-3.5775\;*\;x1\_norm\;)-(2.6661\;*\;x2\_norm)+4.7259\;)\\ h5=tanh(\;(-0.2553\;*\;x1\_norm\;)+(\;2.4331\;*\;x2\_norm\;)-4.3883\;) \end{array}\right.$$

$$\;\left\{\begin{array}{c} \varepsilon^{\prime }=\left(3.3787*h1\right)-\left(5.2749*h2\right)-\left(6.6964*h3\right)-\left(2.8869*h4\right)-(5.6468*h5)+7.5727\\ \varepsilon^{\prime \prime }=\left(\;3.6003*h1\right)-\left(1.0600*h2\right)-\left(1.1128*h3\right)-\left(0.6040*h4\right)-\left(0.7204*h5\right)+1.0966 \end{array}\right.$$

Table 6 presents the machine learning (MLP) calibration results for sandy soil, reporting the predicted real and imaginary permittivity components across different moisture levels along with their corresponding sensor responses.

To evaluate the accuracy of the proposed soil sensor and the various proposed calibration equations, the mean absolute error (MAE) and root mean square error (RMSE) were selected. The coefficient of determination (R^2^) was used to evaluate the degree of fit of the calibration equations.

- Root mean square error (RMSE): The RMSE shows how far predictions fall from measured true values using Euclidean distance. The values closer to 0 indicate better fitting models.

(13) $$RMSE=\sqrt{\frac{\sum_{i=1}^{n}{\left(p_{i}-M_{i}\right)}^{2}}{n}}$$

- Mean absolute error (MAE): Mean absolute error (MAE) is a metric that calculates the average magnitude of the absolute errors between the predicted and actual values. The closer the MAE value to zero, the better the prediction.

(14) $$MAE=\frac{1}{n}\sum_{i=1}^{n}\left|M_{i}-P_{i}\right|$$

- Coefficient of determination (R^2^): It indicates the extent of predictability (i.e., how well dependent variables can be accurately predicted). A value closer to 1 indicates a high level of prediction (model performance).

(15) $$R^{2}=1-\frac{\sum_{i=1}^{n}{\left(M_{i}-P_{i}\right)}^{2}}{\sum_{i=1}^{n}{\left(M_{i}-\bar{M}\right)}^{2}}$$

“n” is the sample size; “$M_{i}$” is the measured value for the ith observation in the dataset; “$P_{i}$” is the predicted value for the “$i^{th}$” observation in the dataset; and “$\bar{M}$” is the mean of the measured values.

To objectively assess the calibration performance of the proposed approaches, three modeling strategies were implemented and compared: (i) a linear regression model, (ii) nonlinear polynomial/least-squares models, and (iii) a machine learning-based model. Table 7 summarizes their mean absolute errors (MAE), root mean square errors (RMSE) and coefficients of determination (R^2^). The results show that the linear model exhibits the largest errors and the lowest (R^2^), confirming that it cannot adequately capture the nonlinear behavior of the sensor response. Introducing nonlinearity through polynomial and least-squares fitting significantly reduces MAE and RMSE, yielding (R^2^) values close to unity. Nonetheless, the machine learning calibration achieves the smallest errors and the highest (R^2^), indicating a quasi-perfect agreement with the reference measurements and making it the most accurate calibration strategy among the three methods.

It is important to note that the near ideal MAE, RMSE, and R^2^ values reported in Table 7 were obtained on the same discrete moisture–permittivity points that were used to train the MLP models. Because only seven (sand) and nine (loam) calibration points were available from the full wave simulations, no statistically meaningful separation into training, validation, and independent test sets was possible. Consequently, the reported metrics quantify the interpolation capability of the MLP within the calibrated range rather than true extrapolative prediction on completely unseen data. Owing to the limited number of available calibration points, the MLP architecture (two inputs, five hidden neurons, and two outputs) contains more trainable parameters than data samples. This inevitably increases the risk of overfitting or memorization if the network is interpreted as a general purpose predictor. In this study, the MLP is therefore deliberately used as a compact nonlinear function approximator over a narrow range of operating conditions, with strong regularization of the input space through the “delta” representation and z score standardization.

### 3.2. Empirical Modeling Techniques for Extracting Loam Soil Complex Permittivity

For the loam soil, the proposed sensor demonstrates an FDR of 6.86 MHz. This indicates a strong resolution capability, allowing the sensor to accurately isolate subtle variations in soil moisture.

**Figure 5:**
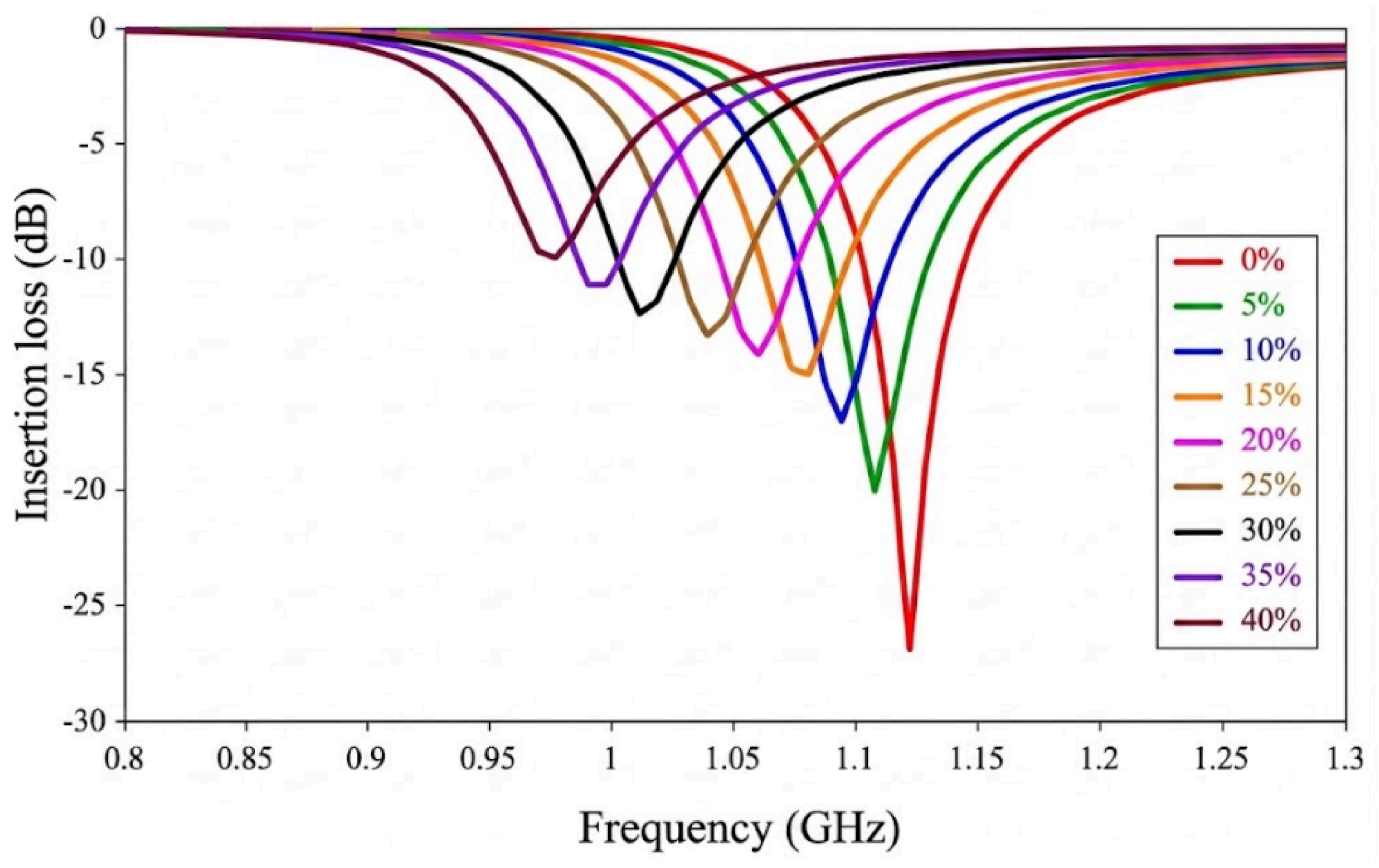
S_21_ frequency shifts with various moisture levels of loam soil samples.

The sensitivity of the designed square CSRR-based sensor is evaluated using the same procedure described in Section 3.1, by analyzing variations in its resonance frequency (Figure 5). Table 8 reports the sensitivity of the CSRR-based soil moisture sensor for loam soil.

#### 3.2.1. Linear and Polynomial Fitting Model of Loam Soil

**Figure 6:**
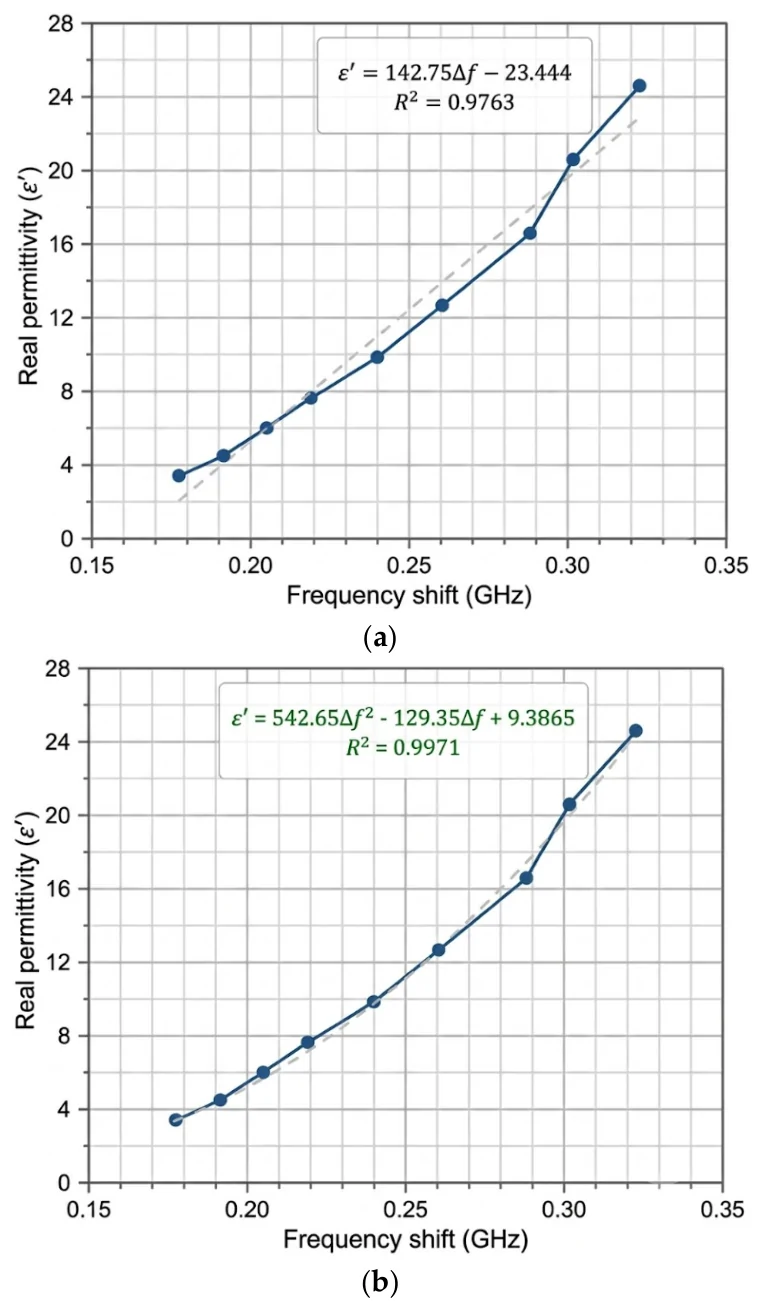
Curve fitting for real permittivity vs. frequency shift: (**a**) linear model, and (**b**) polynomial model.

For the loam soil, Figure 6 illustrates the curve fitting between the real permittivity and the resonance frequency shift using both linear and polynomial models. Similar to the sand case, the linear regression in Figure 6a assumes a first-order dependence, whereas Figure 6b employs a second-order polynomial to capture the nonlinear behavior of permittivity with respect to frequency shift over the 0–40% MC range. The polynomial order was again determined using a leave-one-out cross-validation procedure on the nine available loam calibration points. The validation error decreased significantly when moving from the linear model (first order) to a second-order polynomial, while higher-order polynomials (order ≥ 3) led to increased cross-validated error and non-physical oscillations between the calibration points, indicative of overfitting. Therefore, a second-order polynomial was selected as the most appropriate compromise between model flexibility and robustness for loam soil.

Table 9 presents the corresponding fitting results, listing the resonance frequency, simulated real permittivity, and predicted values from the linear and second-order polynomial models. Both models capture the overall increase in permittivity with moisture content, but the second-order polynomial yields predictions that are systematically closer to the simulated data, especially at intermediate and higher moisture levels. This confirms that the adopted second-order polynomial model provides a more accurate calibration of the sensor response for loam soil.

#### 3.2.2. Least-Squares Model of Loam Soil

As detailed previously for the sand soil, the loam soil calibration also employs a multivariable least-squares sensitivity-matrix approach to retrieve both the real (ε′) and imaginary (ε′) parts of the complex permittivity. The same concept is applied here: resonance frequency shift Δf and insertion-loss shift $\Delta S_{21}$ are jointly related to ε′ and ε″ using experimentally obtained calibration data (Table 10 and Table 11), with 20% MC selected as the reference condition.

The resulting sensitivity matrix is inverted to obtain operational prediction formulas for ∆ε′ and ∆ε″ directly from measured Δf and $\Delta S_{21}$. The predicted complex permittivity values for loam soil (Table 12) show good agreement with the simulated reference data over the 0–40% MC range, confirming that the least-squares model can reliably reconstruct both real and imaginary components. As in the sand case, slightly larger deviations are observed at higher moisture contents, where dielectric losses and soil heterogeneity become more pronounced.

- Predicting Real Permittivity (ε′)

$$\varepsilon^{\prime }=-187.7423\;∆f+0.4678{∆S}_{21}+9.8$$

- Predicting Imaginary Permittivity ($\varepsilon^{\prime \prime }$)

$$\;\varepsilon^{\prime \prime }=-17.2531∆f-0.0156{∆S}_{21}+1.2$$

#### 3.2.3. Machine Learning Model of Loam Soil

Similarly, the machine learning-based calibration adopted for sand soil is extended to loam soil without changing the overall framework. A multilayer perceptron (MLP) is trained using the same “delta” representation, in which the 20% MC operating point is used as the reference and mapped to ((0, 0)) in both input (frequency and magnitude shifts) and output (changes in (ε′) and (ε″) domains). The two input features (Δf and $\Delta S_{21}$) are again standardized via Z-score normalization.

The MLP architecture (a two-neuron input layer, one hidden layer with five tanh neurons, and a two-neuron linear output layer) is preserved. After training on the loam dataset, the network predictions reported in Table 13 accurately follow the evolution of both real and imaginary permittivity across 0–40% MC, with errors systematically lower than those of the polynomial and least-squares models. When trained separately on the sand and loam datasets, the MLP accurately fits the nonlinear relationship between the measured frequency and magnitude shifts and the complex permittivity for each investigated soil texture. Because the present study trains and evaluates distinct MLP models for sand and loam using texture-specific calibration data, it does not yet demonstrate cross-texture generalization in which a single model would be applied to multiple soil types; demonstrating such cross-texture generalization would require a larger and more diverse multi-texture dataset and is left for future work. 

A quantitative comparison of the calibration models for loam soil, in terms of MAE, RMSE, and R^2^, is presented in Table 14.

In the present work, the experimental soil-loaded measurements (Section 4.3, Figures 11 and 12 and Tables 17 and 18) are therefore used to qualitatively verify the consistency between the measured S parameter trends and the simulated calibration curves, rather than to claim fully independent test set performance. Future work will focus on collecting substantially larger experimental datasets, including repeated measurements over finer moisture increments, to enable the rigorous separation of training, validation, and test sets and to quantify the generalization performance of the MLP on completely independent experimental samples. We explicitly acknowledge that, under these conditions, the excellent agreement between MLP predictions and reference permittivity values primarily reflects the model’s ability to interpolate a smooth mapping between frequency/magnitude shifts and complex permittivity within the calibrated range, rather than demonstrating broad generalization to arbitrary soils and moisture conditions. Accordingly, the results are interpreted as a proof of concept for data-driven calibration with limited data, and not as evidence that the present MLP configuration is immune to overfitting. Collection of larger, more diverse datasets and systematic regularization and cross-validation will be required to rigorously demonstrate genuine predictive capability.

## 4. Experimental Prototype and Validation

### 4.1. Sensor Fabrication

The proposed square CSRR-based sensor was fabricated using standard PCB technology on Rogers RO3010 substrate with thickness = 1.27 mm, and 35 µm copper cladding on both sides. The layout strictly follows the optimized dimensions in Figure 1 to preserve the designed resonance characteristics and field confinement in the sensing region. Surface-mount connectors were used to interface the input and output ports with the vector network analyzer (VNA_ E5071B).

**Figure 7:**
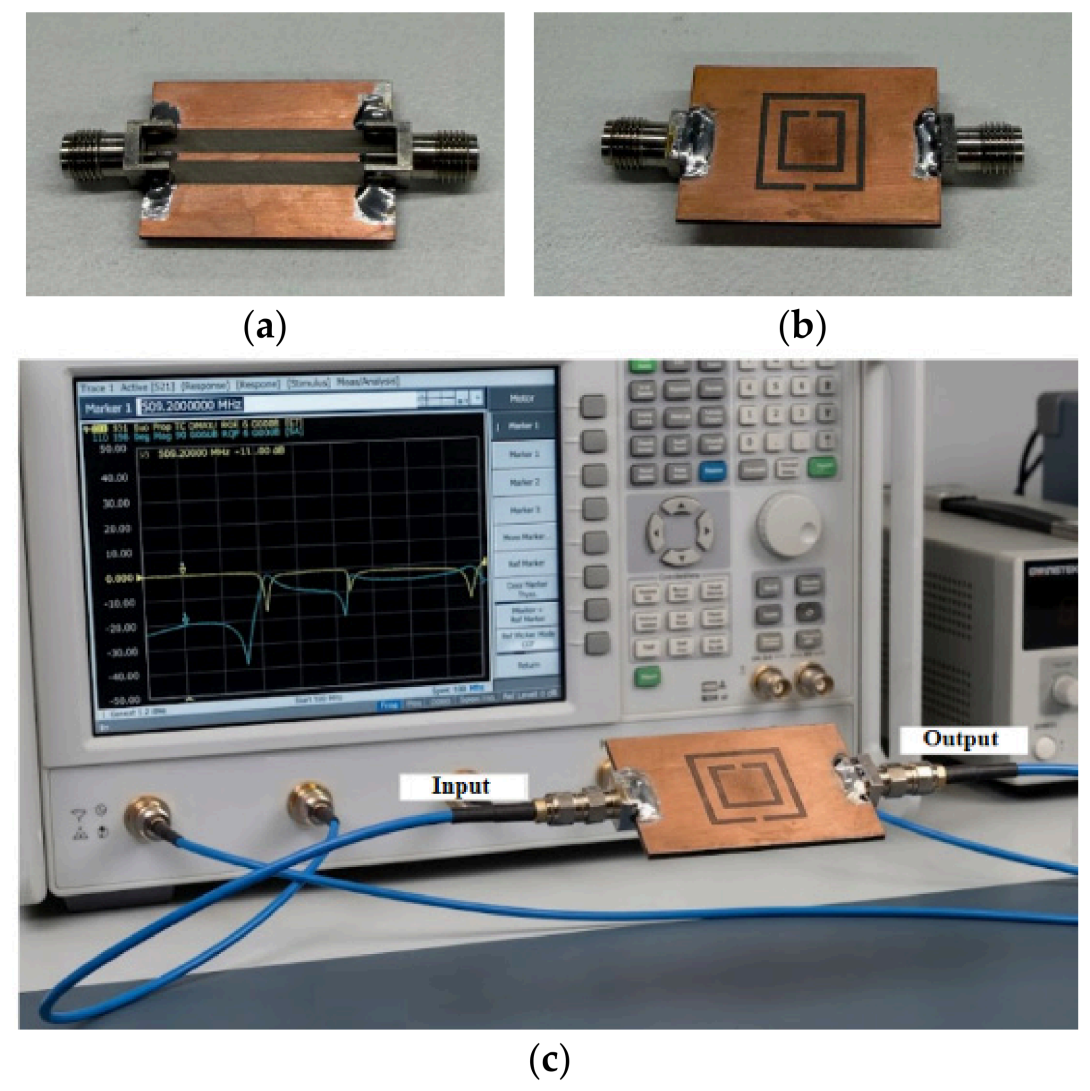
Photographs of the fabricated square SRR-based soil moisture sensor prototype: (**a**) top view showing the square SRR and T-shaped feedline, (**b**) bottom view of the ground plane, and (**c**) measurement setup with VNA.

**Figure 8:**
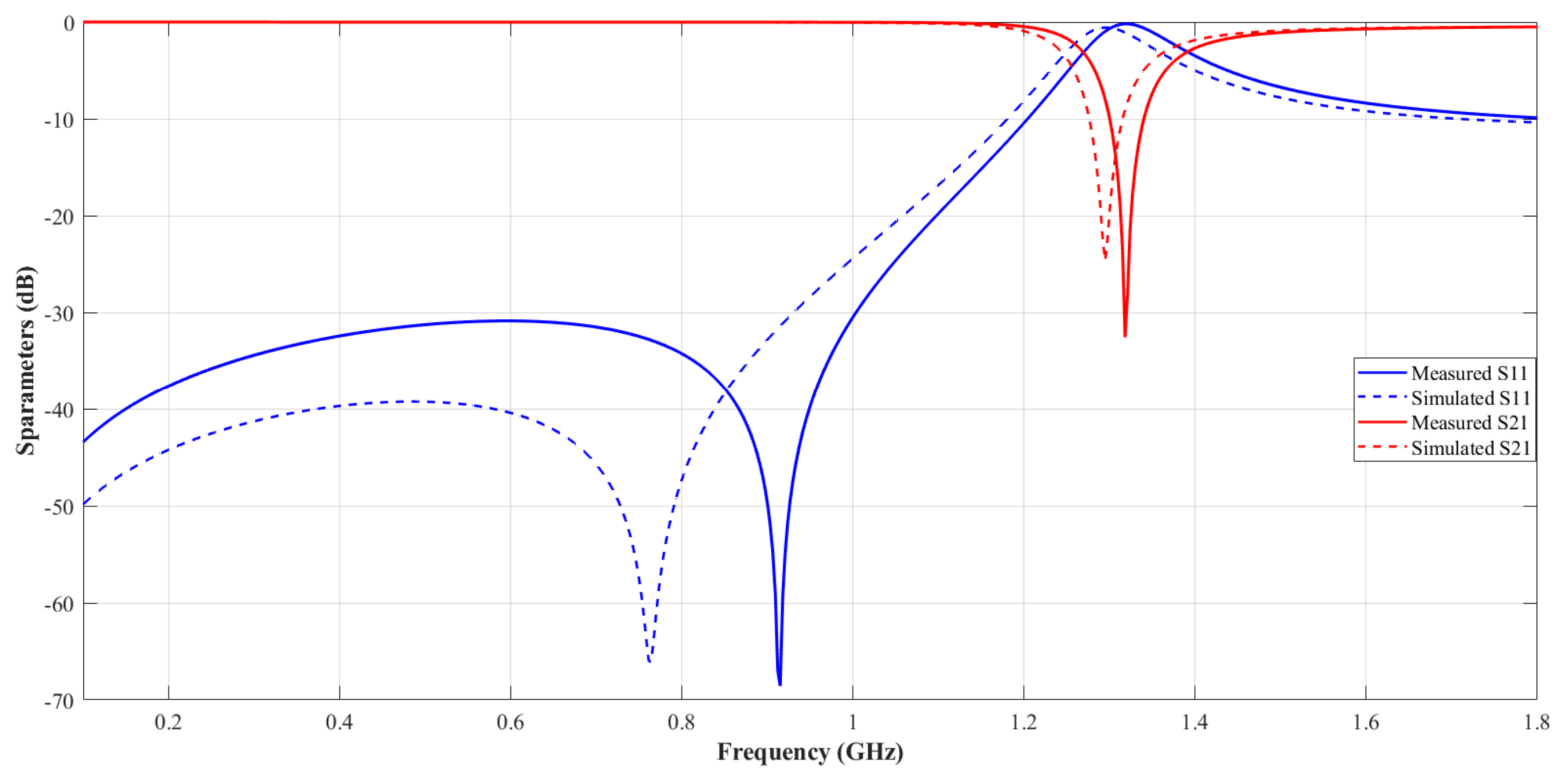
Simulated and measured |S11| and |S21| response of the proposed square CSRR-based sensor.

Figure 7 shows photographs of the fabricated prototype, including (a) the top view with the square CSRR and T-shaped feedline, (b) the bottom ground plane, and (c) a close-up of the sensing region. The measured and simulated |S11| and |S21| responses of the unloaded sensor (without soil) are compared in Figure 8. The fabricated device exhibits a clear resonance around 1.3 GHz, in good agreement with simulations, with minor deviations in resonance frequency and insertion loss that can be attributed to fabrication tolerances, connector parasitics, and slight discrepancies in the actual substrate permittivity and loss tangent.

### 4.2. Measurement Setup

**Figure 9:**
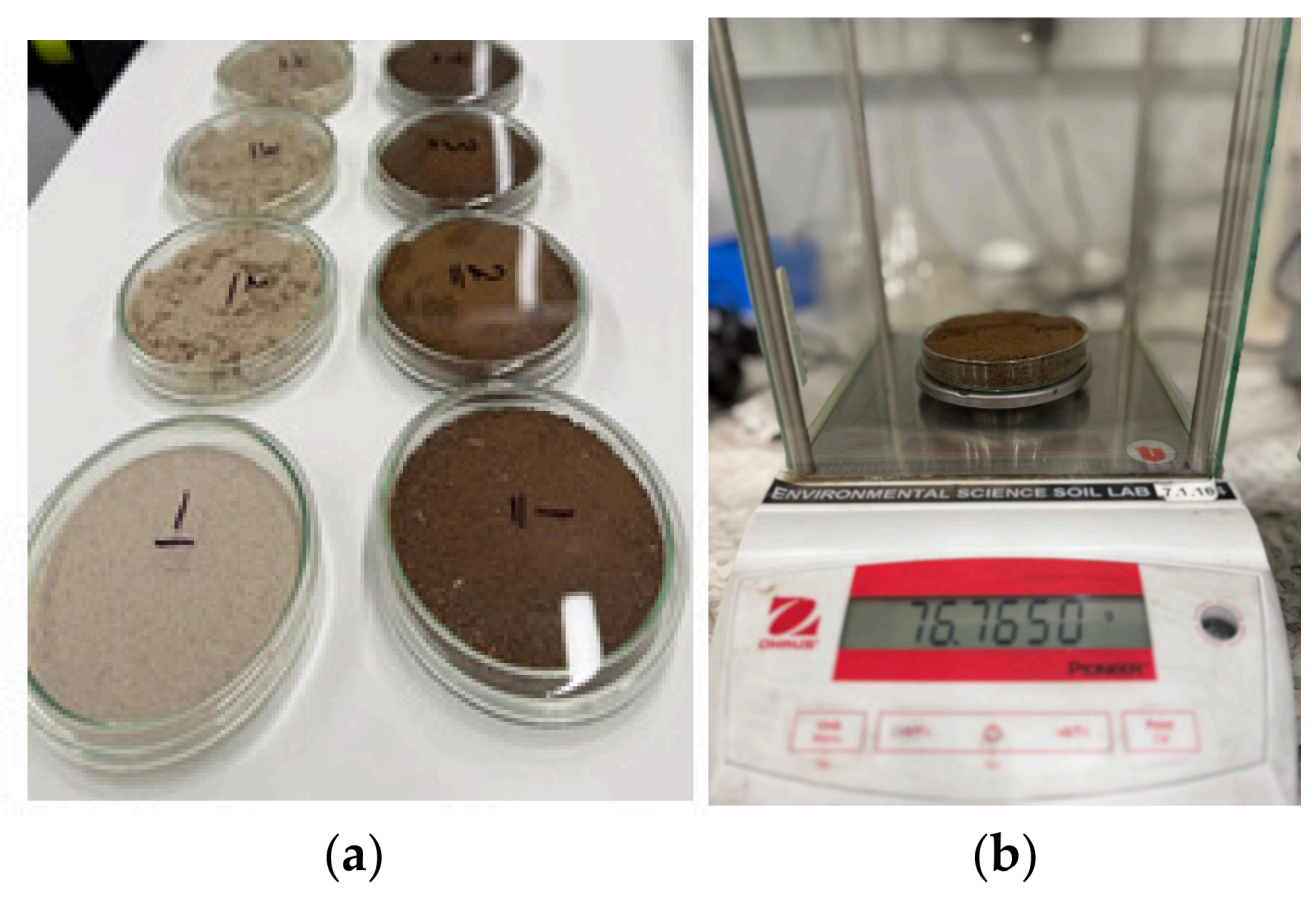
Soil preparation: (**a**) soil samples, and (**b**) gravimetric moisture content procedure.

Two soil types were selected for experimental validation: sandy soil and loamy soil (Figure 9). Bulk soil samples were first air-dried at room temperature, gently crushed, and sieved to remove stones and large organic debris, ensuring homogeneous packing in the soil box. The gravimetric moisture content (MC) was adjusted by adding predetermined amounts of water to the dry soil. For each target MC level, the soil mass before and after water addition was recorded using a precision balance (±0.0001 g) to verify the actual moisture content. The conditioned samples were then sealed in airtight containers and allowed to rest for several hours to promote uniform redistribution of water prior to measurement.

**Figure 10:**
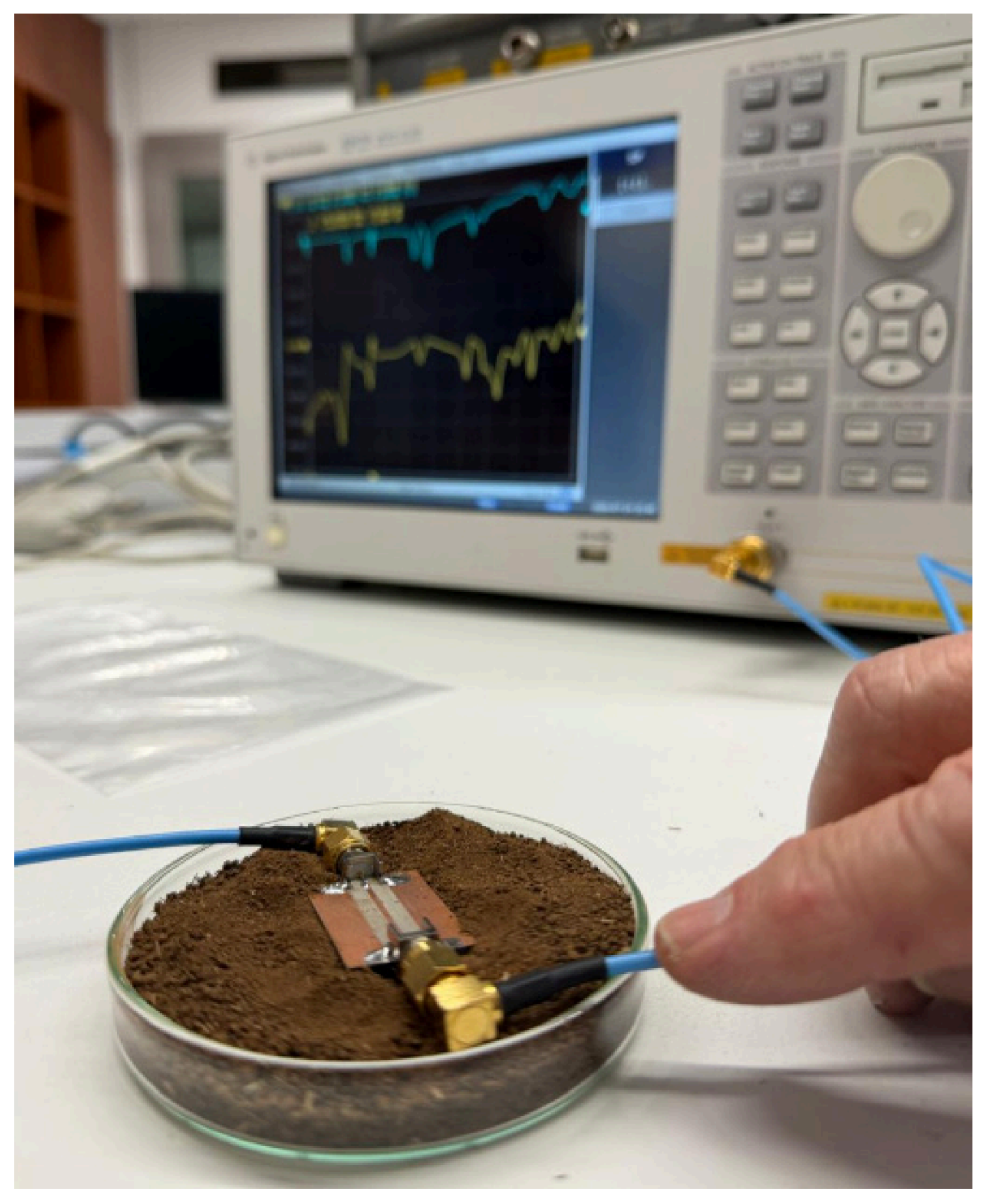
Measurement setup of the sensor with the soil sample.

To ensure reproducible interaction between the electromagnetic field and the Soil Under Test (SUT), a dedicated soil container was used under the CSRR, as shown in Figure 10.

The measurement procedure comprised the following steps. First, subsamples of dry soil were precisely weighed and the required amount of water was added to reach the desired MC level. The moist soil was placed into labeled containers and its frequency response (resonance frequency and insertion loss) was measured using the proposed sensor integrated into a stabilized holder to minimize movement during VNA measurements. After the microwave measurements, each sample was transferred to a preheated oven and dried at 105–110 °C for 24 h to determine the final dry mass.

The gravimetric moisture content was then calculated using:

(16) $$MC\left(\%\right)=\frac{m_{water}}{m_{DrySoil}}\times 100\%=\frac{M_{2}-M_{3}}{M_{3}-M_{1}}\times 100\%$$

where M_1_ is the mass of the empty container, M_2_ is the mass of the container plus moist soil, and M_3_ is the mass of the container plus oven-dried soil. This protocol was applied to establish experimental calibration points for both sandy and loamy soils. The measured weights and corresponding moisture contents are summarized in Table 15 and Table 16.

To control soil bulk density and compaction, all samples were prepared following a consistent packing protocol. After adjusting the gravimetric moisture content, the soil was gently poured into the container in three equal layers, and each layer was leveled and lightly tapped a fixed number of times using the same tool to achieve reproducible compaction. The mass of soil in the container was kept constant for each moisture group, so that the bulk density remained approximately constant across moisture levels for a given soil texture.

All measurements were carried out in an air-conditioned laboratory. Deionized water was used for wetting, and the initial electrical conductivity of the soils was low, so that salinity effects are expected to be negligible. Particle size distribution was controlled by sieving and classification into sand and loam textures as described in Section 3, and the container geometry and fill height were kept constant so that the sample volume exposed to the near field of the CSRR remained unchanged. The sensor was placed directly on top of the soil surface with gentle pressure to ensure intimate contact and to minimize air gaps at the sensor–soil interface.

### 4.3. Measured Soil-Loaded Response and Model Validation

**Figure 11:**
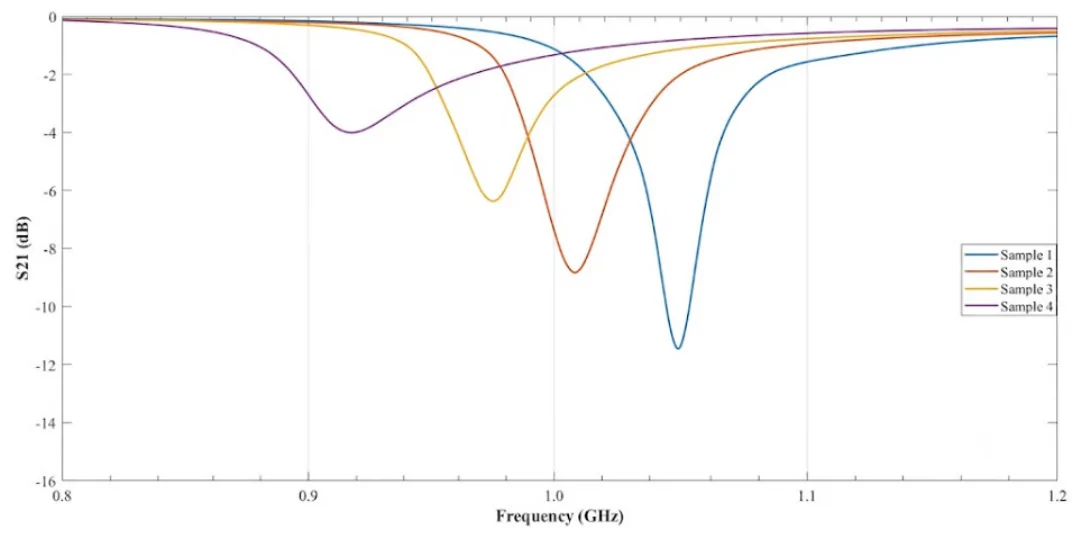
Measured S_21_ response of the square CSRR-based sensor with sandy soil.

**Figure 12:**
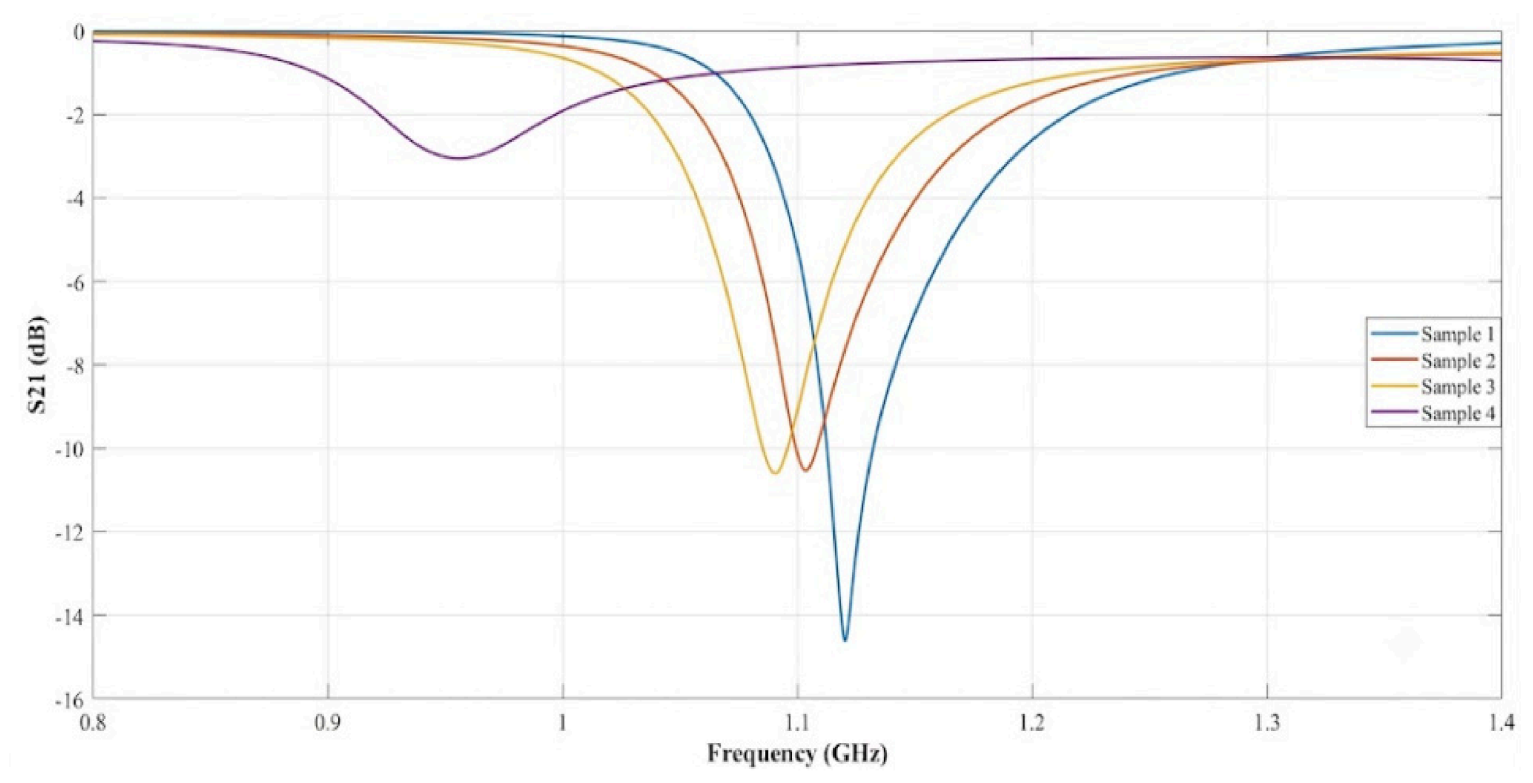
Measured S_21_ response of the square CSRR-based sensor with loamy soil.

The measured transmission responses (S21) of the square CSRR-based sensor loaded with sandy and loamy soils at different moisture contents are shown in Figure 11 and Figure 12, respectively. For each soil type, four representative moisture groups were considered, spanning practical agricultural ranges. The corresponding resonance frequencies, insertion losses, and gravimetrically determined moisture contents are listed in Table 17 for sandy soil and Table 18 for loamy soil.

The measured resonance frequencies, insertion losses, and corresponding gravimetric moisture contents for the fabricated square CSRR-based sensor loaded with sandy soil are summarized in Table 17.

The analogous measured performance of the sensor for loamy soil, including resonance frequency, insertion loss, and gravimetric moisture content for each measurement group, is reported in Table 18.

In this work, the gravimetric measurements described in Section 4.2 are used to determine the actual moisture content of each prepared soil sample and to verify the monotonic relationship between moisture content and the sensor’s resonant frequency and insertion loss. However, a fully closed validation loop—where predicted complex permittivity is converted to volumetric or gravimetric moisture and quantitatively compared with independent gravimetric and dielectric reference measurements, including detailed uncertainty propagation—has not yet been implemented.

The current results should therefore be viewed as an initial validation of the sensing principle and calibration framework under controlled laboratory conditions, rather than a complete metrological assessment. Future work will integrate an independent dielectric measurement system (e.g., coaxial probe or resonant cavity) to obtain reference complex permittivity values, and will explicitly propagate uncertainties from VNA measurements, calibration model parameters, and gravimetric measurements to quantify the overall moisture prediction uncertainty and confidence intervals.

## 5. Discussion

### 5.1. Regression Model

The regression models developed for both sand and loam soils effectively correlate the sensor’s resonance frequency shifts with soil moisture content, demonstrating strong predictive accuracy. Polynomial fitting models outperform linear models, as evidenced by higher R^2^ values (0.9991 for sand and 0.9971 for loam), highlighting their ability to capture the inherent nonlinear relationship between soil permittivity and moisture. The least-squares model advances this by incorporating both resonance frequency and insertion loss, allowing extraction of the complex permittivity’s real and imaginary components. This dual-parameter approach is critical for accounting for dielectric losses that influence sensor behavior, thereby improving moisture content estimation accuracy. The machine learning model, specifically the multilayer perceptron (MLP), achieves the highest precision with minimal MAE and RMSE, and R^2^ values near unity. This confirms its superior capacity to model complex nonlinearities and sensor response patterns beyond the scope of empirical fitting, leveraging data-driven learning for robust soil moisture prediction.

### 5.2. Error Analysis

The error analysis clearly indicates that the machine learning model delivers the most accurate soil moisture predictions, with MAE and RMSE values significantly lower than those of the empirical models across both sand and loam soils. For sand soil, the machine learning model achieved an MAE of 0.0005, RMSE of 0.0007, and R^2^ of 0.999, while for loam soil, these values were 0.0007, 0.0009, and 0.999 respectively. The linear regression model exhibits the highest error rates, with MAE and RMSE values of 0.6336 and 0.6779 (R^2^ = 0.9845) for sand, and 0.9321 and 1.0739 (R^2^ = 0.9763) for loam, reflecting its inability to capture the complex nonlinear dielectric behavior of soils effectively. Polynomial and least-squares models improve prediction accuracy by better fitting the nonlinear relationship and incorporating both resonance frequency and insertion loss; however, they still fall short of the machine learning model’s performance. Polynomial fitting models show MAE of 0.1464 and 0.2811, RMSE of 0.1603 and 0.3728, and R^2^ of 0.9991 and 0.9971 for sand and loam respectively, while least-squares models report MAE of 0.1759 and 0.9004, RMSE of 0.2481 and 1.0088, and R^2^ of 0.9979 and 0.9791 respectively. The machine learning approach, through its multilayer perceptron architecture, adapts to complex nonlinear sensor responses and optimizes prediction accuracy, achieving R^2^ values close to 1 and minimal absolute and root mean square errors. The consistently low error margins across varying moisture contents demonstrate the sensor’s high frequency detection resolution and sensitivity—6.09 MHz for sand and 6.86 MHz for loam—confirming its capability for precise moisture quantification. Minor increases in prediction errors at higher moisture levels can be attributed to amplified dielectric losses and soil heterogeneity, suggesting that further refinement through expanded training datasets or enhanced sensor calibration could improve robustness and accuracy.

### 5.3. Comparison with Literature

Compared with existing microwave soil moisture sensors summarized in Table 19, the proposed square CSRR sensor exhibits a distinct combination of operating frequency, footprint, and calibration strategy rather than a uniformly “better” performance. In terms of geometry, its compact size of 20 × 30 mm^2^ facilitates integration in space-constrained environments, while still providing a practically useful sensing volume for local soil characterization. This contrasts with larger planar DGS and cavity-type sensors [19,21], which, although occupying more area (e.g., 50 × 50 mm^2^ and 96 × 46 mm^2^), may interrogate a larger soil volume and therefore offer different trade-offs in terms of spatial representativeness versus local resolution.

From an electromagnetic standpoint, operation near 1.3 GHz differentiates the present design from many reported planar sensors that typically work around 2.4 GHz [19,20,22]. The lower frequency offers a qualitative advantage in terms of penetration depth and robustness to attenuation in wetter soils, at the expense of a modestly larger resonator size. Conversely, higher-frequency designs may provide finer spatial resolution but can be more susceptible to loss in high-moisture or saline conditions. The FDR values obtained in this work (6.09 MHz for sand and 6.86 MHz for loam) indicate high resolution under the specific laboratory conditions and soil textures considered, but they are not directly comparable on an absolute basis with sensitivities reported in other studies due to differences in definitions, soil preparation, and measurement setups.

A further point of differentiation lies in the calibration and interpretation of sensor outputs. Several existing sensors primarily report empirical relations between resonance frequency (or field capacity) and a chosen moisture metric [19,20,21,22], often focusing on the real part of permittivity or on a single scalar moisture indicator. In contrast, the present work explicitly exploits both resonance/notch frequency and insertion-loss magnitude (S21) to recover the complex permittivity (real and imaginary components) of sand and loam. The use of polynomial, least-squares sensitivity-matrix, and MLP-based calibration frameworks enables texture-aware, complex-permittivity-aware characterization over the calibrated ranges. These differences in calibration approach are qualitative and methodological rather than strictly hierarchical: each sensor and model is tailored to its own target application, soil set, and validation protocol.

Overall, the comparison in Table 19 should therefore be interpreted as indicative rather than as a strict ranking of sensor performance. The cited works differ in measurement principle, operating frequency, moisture definition (gravimetric, volumetric, or field capacity), soil types, and validation procedures, which prevents direct numerical benchmarking. Within these limitations, the proposed square CSRR sensor can be viewed as a complementary option that combines a relatively low operating frequency, compact footprint, and data-driven calibration strategies to enable localized, texture-aware soil moisture sensing under controlled conditions. Further multi-site, multi-texture studies would be required to establish comprehensive performance benchmarks across sensor families.

## 6. Conclusions

This work set out to determine whether a compact CSRR-based microwave sensor, when combined with advanced calibration strategies, can provide accurate, texture-aware soil moisture quantification across practical moisture ranges for different soil types. The proposed square CSRR sensor, implemented on a Rogers RO3010 substrate and operating near 1.3 GHz within a 20 × 30 mm^2^ footprint, demonstrated high frequency detection resolution and sensitivity for both sand and loam soils, with FDR values of 6.09 MHz and 6.86 MHz, respectively. By exploiting simultaneous shifts in resonance/notch frequency and insertion loss, the sensor was shown to be capable of resolving subtle changes in the real and imaginary components of soil permittivity over 0–30% MC (sand) and 0–40% MC (loam).

A systematic comparison of calibration approaches—linear and polynomial regression, a multivariable least-squares sensitivity-matrix model, and a machine-learning-based multilayer perceptron (MLP)—confirmed that simple linear fits are insufficient to capture the strongly nonlinear, texture-dependent dielectric behavior of soils. Polynomial and least-squares models significantly improved accuracy (R^2^ > 0.997), particularly by jointly using frequency and magnitude information to recover complex permittivity. However, the MLP-based calibration consistently delivered near-ideal performance (R^2^ ≈ 1 with MAE and RMSE < 0.001), robustly reconstructing both real and imaginary permittivity for sand and loam and thereby offering the most reliable moisture estimates. These findings, together with the compact footprint and operation at a relatively low microwave frequency, position the proposed CSRR sensor as a competitive alternative to existing planar and cavity-based microwave soil moisture sensors.

Beyond the immediate experimental results, this work highlights the broader potential of combining high-Q metamaterial-inspired resonators with data-driven calibration to build compact, accurate, and texture-aware soil moisture sensing platforms. Such systems can underpin precision irrigation scheduling, improved field-scale water balance assessment, and enhanced geotechnical risk monitoring, ultimately contributing to more sustainable agricultural water use and better management of soil-related infrastructure hazards.

Future research will focus on extending the approach along several directions. First, validation will be expanded to a wider range of soil textures, salinities, temperatures, and bulk densities to assess model robustness under realistic field variability. Second, larger and more diverse datasets will be collected to train and test more sophisticated machine-learning architectures (e.g., ensembles or deeper networks) and to investigate transfer learning between soil types. Third, the CSRR sensor will be integrated into autonomous, multi-sensor field platforms, including wireless nodes, to enable long-term in situ monitoring and data fusion with other agronomic and environmental measurements.

## Figures and Tables

**Table 1:** Sensitivity of the sand soil moisture sensor.

Moisture Level (MC)	ε′ Dataset [23]	$\varepsilon^{\prime \prime }$ Dataset [23]	$f_{notch}$ (GHz) Simulated	$∆f_{notch}$ (GHz) Simulated	S21 (dB) Simulated	$S_{n}$ (%)
0%	3.4	0.0	1.0425	0.2575	23.728	8.2532
5%	4.5	0.1	1.0338	0.2662	21.516	5.8505
10%	6.2	0.3	1.0164	0.2836	18.305	4.1953
15%	8.8	0.6	0.999	0.301	16.128	2.9684
20%	12	0.7	0.9816	0.3184	15.777	2.2266
25%	15.6	1.0	0.9642	0.3358	14.368	1.7692
30%	19.1	1.3	0.9468	0.3532	13.344	1.5011

**Table 2:** Curve-fitting model results.

Curve-Fitting Models	Moisture Level (MC)	ε′ Dataset [23]	$∆f_{notch}$ (GHz) Simulated	ε′ Simulated	Error (%)
**Linear Model**	**0%**	3.4	0.2575	2.6179	23.003
	**5%**	4.5	0.2662	4.0423	10.172
	**10%**	6.2	0.2836	6.891	11.145
	**15%**	8.8	0.301	9.7397	10.679
	**20%**	12	0.3184	12.588	4.9037
	**25%**	15.6	0.3358	15.437	1.0437
	**30%**	19.1	0.3532	18.286	4.2623
**Polynomial Model**	**0%**	3.4	0.2575	3.4356	1.0484
	**5%**	4.5	0.2662	4.308	4.266
	**10%**	6.2	0.2836	6.3922	3.0992
	**15%**	8.8	0.301	8.9287	1.4629
	**20%**	12	0.3184	11.918	0.68516
	**25%**	15.6	0.3358	15.359	1.543
	**30%**	19.1	0.3532	19.253	0.80242

**Table 3:** Calibration results for the sandy soil sensor output model.

Moisture Level MC	ε′	ε″	∆ε′	∆ε″
0%	3.4	0.0	−5.4	−0.6
5%	4.5	0.1	−4.3	−0.5
10%	6.2	0.3	−2.6	−0.3
15%	8.8	0.6	0.0	0.0
20%	12	0.7	3.2	0.1
25%	15.6	1.0	6.8	0.4
30%	19.1	1.3	10.3	0.7

**Table 4:** Calibration results for the sandy soil sensor input model.

Moisture Level	$f_{notch}$ (GHz)	$S_{21}$ (dB)	∆f (GHz)	${∆S}_{21}$ (dB)
0%	1.0425	23.728	0.0435	7.6
5%	1.0338	21.516	0.0348	5.388
10%	1.0164	18.305	0.0174	2.177
15%	0.999	16.128	0	0
20%	0.9816	15.777	−0.0174	−0.351
25%	0.9642	14.368	−0.0348	−1.76
30%	0.9468	13.344	−0.0522	−2.784

**Table 5:** Summary of least-squares calibration model results for sandy soil.

Moisture Level (MC)	ε′ Dataset [23]	ε″ Dataset [23]	∆f (GHz)	${∆S}_{21}$ (dB)	ε′ Simulated	ε″ Simulated	Error (%) ε′	Error (%) ε″
0%	3.4	0.0	0.0435	7.6	3.49	0.068	2.63	NaN
5%	4.5	0.1	0.0348	5.388	4.148	0.084	7.81	15.78
10%	6.2	0.3	0.0174	2.177	6.173	0.356	0.43	18.69
15%	8.8	0.6	0	0	8.8	0.6	0	0
20%	12	0.7	−0.0174	−0.351	12.491	0.795	4.09	13.52
25%	15.6	1.0	−0.0348	−1.76	15.565	1.018	0.22	1.79
30%	19.1	1.3	−0.0522	−2.784	18.864	1.231	1.24	5.33

**Table 6:** Machine Learning model results.

Moisture Level (MC)	ε′ Dataset [23]	ε″ Dataset [23]	∆f (GHz)	${∆S}_{21}$ (dB)	ε′ Simulated	ε″ Simulated	Error (%) ε′	Error (%) ε″
0%	3.4	0.0	0.0435	7.6	3.3999	0.0004	0.01	N/A
5%	4.5	0.1	0.0348	5.388	4.5000	0.0995	0.01	0.48
10%	6.2	0.3	0.0174	2.177	6.1999	0.3005	0.01	0.18
15%	8.8	0.6	0	0	8.8003	0.5989	0.01	0.19
20%	12	0.7	−0.0174	−0.351	12.0006	0.6965	0.01	0.49
25%	15.6	1.0	−0.0348	−1.76	15.5987	1.0096	0.01	0.96
30%	19.1	1.3	−0.0522	−2.784	19.1009	1.2950	0.01	0.39

**Table 7:** Summarized statistics of sensor accuracy of sand soil.

Calibration Model	Progression Equation	MAE	RMSE	R^2^
Linear Model	$\varepsilon^{\prime }=163.72{∆f}_{notch}-39.54$	0.6336	0.6779	0.9845
Polynomial Model	$\varepsilon^{\prime }=747.23{{∆f}_{notch}}^{2}-291.05{∆f}_{notch}+28.835$	0.1464	0.1603	0.9991
Least-Squares	$\varepsilon^{\prime }=-223.8669∆f+{0.5826∆S}_{21}+8.8$	0.1759	0.2481	0.9979
	$\varepsilon^{\prime \prime }=-10.64∆f-0.027{∆S}_{21}+0.6$	0.0460	0.0562	0.9837
Machine Learning	$\varepsilon^{\prime }=\left(3.3787*h1\right)-\left(5.2749*h2\right)-\left(6.6964*h3\right)-\left(2.8869*h4\right)-(5.6468*h5)+(7.5727)$	0.0005	0.0007	1
	$\varepsilon^{\prime \prime }=\left(3.6003*h1\right)-\left(1.0600*h2\right)-\left(1.1128*h3\right)-\left(0.6040*h4\right)-(0.7204*h5)+\;1.0966$	0.0029	0.0043	0.999

**Table 8:** Sensitivity of the loam soil moisture sensor.

Moisture Level (MC)	ε′ Dataset [23]	ε″ Dataset [23]	$f_{notch}$ (GHz) Simulated	$∆f_{notch}$ (GHz) Simulated	S21 (dB) Simulated	$S_{n}$ (%)
0%	3.4	0.0	1.1212	0.1788	26.842	5.7308
5%	4.5	0.3	1.1074	0.1926	19.978	4.233
10%	6.0	0.6	1.0936	0.2064	16.965	3.1754
15%	7.6	0.9	1.0798	0.2202	14.951	2.5664
20%	9.8	1.2	1.0591	0.2409	14.063	2.1058
25%	12.6	1.5	1.0384	0.2616	13.269	1.7347
30%	16.5	1.8	1.0108	0.2892	12.295	1.4352
35%	20.5	2.3	0.997	0.303	11.075	1.1953
40%	24.5	2.9	0.9763	0.3237	9.9107	1.0596

**Table 9:** Summary of curve-fitting model performance for loamy soil sensing.

Curve-Fitting Models	Moisture Level (MC)	ε′ Dataset [23]	$∆f_{notch}$ (GHz) Simulated	ε′ Simulated	Error (%)
**Linear Model**	0%	3.4	0.1788	2.0797	38.832
	5%	4.5	0.1926	4.0496	10.008
	10%	6.0	0.2064	6.0196	0.3267
	15%	7.6	0.2202	7.9896	5.1257
	20%	9.8	0.2409	10.944	11.678
	25%	12.6	0.2616	13.899	10.313
	30%	16.5	0.2892	17.839	8.117
	35%	20.5	0.303	19.809	3.3695
	40%	24.5	0.3237	22.764	7.085
**Polynomial Model**	0%	3.4	0.1788	3.6069	6.0864
	5%	4.5	0.1926	4.6032	2.2925
	10%	6.0	0.2064	5.8061	3.2322
	15%	7.6	0.2202	7.2157	5.0570
	20%	9.8	0.2409	9.7176	0.8409
	25%	12.6	0.2616	12.685	0.6711
	30%	16.5	0.2892	17.364	5.2358
	35%	20.5	0.303	20.014	2.3727
	40%	24.5	0.3237	24.376	0.5074

**Table 10:** Calibration results for the loamy soil sensor output model.

Moisture Level	ε′	ε″	∆ε′	∆ε″
0%	3.4	0.0	−6.4	−1.2
5%	4.5	0.3	−5.3	−0.9
10%	6.0	0.6	−3.8	−0.6
15%	7.6	0.9	−2.2	−0.3
20%	9.8	1.2	0	0
25%	12.6	1.5	2.8	0.3
30%	16.5	1.8	6.7	0.6
35%	20.5	2.3	10.7	1.1
40%	24.5	2.9	14.7	1.7

**Table 11:** Calibration results for the loamy soil sensor input model.

Moisture Level	$f_{notch}$ (GHz)	$S_{21}$ (dB)	∆f (GHz)	${∆S}_{21}$ (dB)
0%	1.1212	26.842	0.0621	12.779
5%	1.1074	19.978	0.0483	5.915
10%	1.0936	16.965	0.0345	2.902
15%	1.0798	14.951	0.0207	0.888
20%	1.0591	14.063	0	0
25%	1.0384	13.269	−0.0207	−0.794
30%	1.0108	12.295	−0.0483	−1.768
35%	0.997	11.075	−0.0621	−2.988
40%	0.9763	9.9107	−0.0828	−4.1523

**Table 12:** Summary of least-squares calibration model results for loamy soil.

Moisture Level (MC)	ε′ Dataset	ε″ Dataset	∆f (GHz)	${∆S}_{21}$ (dB)	ε′ Simulated	ε″ Simulated	Error (%) ε′	Error (%) ε″
0%	3.4	0.0	0.0621	12,779	4.12	0.071	21.17	NaN
5%	4.5	0.3	0.0483	5.915	3.499	0.274	22.24	8.56
10%	6.0	0.6	0.0345	2.902	4.681	0.559	21.99	6.76
15%	7.6	0.9	0.0207	0.888	6.329	0.829	16.72	7.89
20%	9.8	1.2	0	0	9.8	1.2	0.00	0.00
25%	12.6	1.5	−0.0207	−0.794	13.315	1.57	5.67	4.64
30%	16.5	1.8	−0.0483	−1.768	18.041	2.061	9.34	14.50
35%	20.5	2.3	−0.0621	−2.988	20.061	2.318	2.14	0.79
40%	24.5	2.9	−0.0828	−4.1523	23.402	2.693	4.48	7.12

**Table 13:** Summary of machine learning model results for loamy soil.

Moisture Level (MC)	ε′ Dataset	ε″ Dataset	∆f (GHz)	${∆S}_{21}$ (dB)	ε′ Simulated	ε″ Simulated	Error (%) ε′	Error (%) ε″
0%	3.4	0.0	0.0621	12,779	3.4001	0.0000	0.0030	N/A
5%	4.5	0.3	0.0483	5.915	4.5003	0.3000	0.0056	0.0150
10%	6.0	0.6	0.0345	2.902	6.0002	0.6000	0.0036	0.0011
15%	7.6	0.9	0.0207	0.888	7.6005	0.8935	0.0065	0.7213
20%	9.8	1.2	0	0	9.8011	1.2019	0.0115	0.1559
25%	12.6	1.5	−0.0207	−0.794	12.5979	1.5069	0.0170	0.4597
30%	16.5	1.8	−0.0483	−1.768	16.5008	1.7970	0.0046	0.1644
35%	20.5	2.3	−0.0621	−2.988	20.4992	2.3062	0.0038	0.2676
40%	24.5	2.9	−0.0828	−4.1523	24.5001	2.8946	0.0003	0.1876

**Table 14:** Summarized statistics of sensor accuracy of loam soil.

Calibration Model	Progression Equation	MAE	RMSE	R^2^
Linear Model	$\varepsilon^{\prime }=142.75{∆f}_{notch}-23.444$	0.9321	1.0739	0.9763
Polynomial Model	$\varepsilon^{\prime }=542.65{{∆f}_{notch}}^{2}-129.35{∆f}_{notch}+9.3865$	0.2811	0.3728	0.9971
Least-Squares	$\varepsilon^{\prime }=-187.7423\;∆f+0.4678{∆S}_{21}+9.8$	0.9004	1.0088	0.9791
	$\varepsilon^{\prime \prime }=-17.2531∆f-0.0156{∆S}_{21}+1.2$	0.0850	0.1196	0.9821
Machine Learning	$\varepsilon^{\prime }=(15.9861*h1)+(-0.1564*h2)+(-0.6843*h3)+(-4.8956*h4)+(-0.5912*h5)+(15.5852)$	0.0007	0.0009	1
	$\varepsilon^{\prime \prime }=(\;2.4624*h1)+(0.3907*h2)+(0.2800*h3)+(-1.0605*h4)+(-0.1851*h5)+(1.6999)$	0.0033	0.0043	1

**Table 15:** Gravimetric moisture content determination for sandy soil.

Moisture Content Group	Weight of Can, M_1_ (g)	Weight of Can + Wet Soil, M_2_ (g)	Weight of Can + Dry Soil, M_3_ (g)	Calculated MC (%)
1	29.2465	78.9756	77.1721	3.7631
2	29.2979	84.0430	77.2287	14.2169
3	30.8744	89.4140	78.3338	23.3467
4	29.2086	91.6390	77.1023	30.3520

**Table 16:** Gravimetric moisture content determination for loamy soil.

Moisture Content Group	Weight of Can, M_1_ (g)	Weight of Can + Wet Soil, M_2_ (g)	Weight of Can + Dry Soil, M_3_ (g)	Measured MC (%)
1	29.6131	79.5762	79.5530	0.0465
2	29.1885	84.1115	79.3093	9.5813
3	29.6892	86.9140	79.5970	14.6610
4	29.1819	87.3750	77.8857	19.4837

**Table 17:** Measured performances of the fabricated square CSRR-based sensor with sandy soil.

Moisture Content Group	$∆f_{notch}$ (GHz) Measured	$f_{notch}$ (GHz) Measured	ΔS_21_ (dB) Measured	S_21_ (dB) Measured
1	0.25	1.05	20.5	11.5
2	0.29	1.01	23.1	8.9
3	0.33	0.97	25.6	6.4
4	0.38	0.92	28	4.0

**Table 18:** Measured performances of the fabricated square CSRR-based sensor with loamy soil.

Moisture Content Group	$∆f_{notch}$ (GHz) Measured	$f_{notch}$ (GHz) Measured	ΔS_21_ (dB) Measured	S_21_ (dB) Measured
1	0.18	1.12	17.4	14.6
2	0.2	1.10	21.5	10.5
3	0.21	1.09	21.4	10.6
4	0.36	0.94	29	3.0

**Table 19:** Comparison of sensors’ performances.

Ref.	Configuration	Substrate	Operating Frequency (GHz)	Size (mm^2^)	Sensitivity	Soil Type	Moisture Content Range	Measurement Principle	Calibration/Validation Type
[19]	DGS-based planar sensor (mod. CSRR)	Rogers RO3006	2.38	50 × 50	1.83%	Fine sand, Coarse sand, Grit, Clay	7 to 15%	Notch frequency shift with moisture	Laboratory measurements on prepared soil samples; empirical regression
[20]	Self-similar fractal planar sensor (MPS)	FR4	2.4	30 × 30	Not specified	Soil with organic matter content	0–100%	Resonance shift as a function of moisture	Experimental validation over a wide MC range; empirical curve fitting
[21]	Cavity antenna (planar)	Not specified	1.5–3	96 × 46	Not specified	Silt, Clay, and Loam	1–45%	Cavity-type resonant structure; change in cavity response with soil loading	Laboratory measurements on multiple textures; empirical models
[22]	Rotated self-similar fractal (R-SSF) planar sensor	Rogers RT 5880	2.4	60 × 50	Not specified	Sand, Loam, and Clay	0–75% field capacity	Frequency response related to soil field capacity	Experimental validation; empirical calibration per soil type
This work	Square CSRR Sensor	Rogers RO3010	1.3	20 × 30	3.82%	Sand, Loam	0–30% 0–40%	Notch frequency and |S21| magnitude shifts	Combined full-wave simulation and laboratory measurements; polynomial, least-squares and MLP-based calibration models

## Data Availability

The data used and analyzed during the current study are available from the corresponding author on reasonable request.
